# Supplementary material for: A spatially localized DNA linear classifier for cancer diagnosis
Source: Nat Commun. 2024 May 29;15:4583. doi: 10.1038/s41467-024-48869-y (PMC11136972; doi:10.1038/s41467-024-48869-y)
Supplement: Supplementary file 1 — Supplementary Information [file 41467_2024_48869_MOESM1_ESM.pdf]

Supplementary Information

A spatially localized DNA linear classifier for cancer diagnosis

Linlin Yang[1,2,3,5], Qian Tang[1,5], Mingzhi Zhang[2], Yuan Tian[2], Xiaoxing Chen[2], Rui Xu[4], Qian Ma[4], Pei Guo[1]*, Chao Zhang[2,4]*, and Da Han[1,2]*

[1]Zhejiang Cancer Hospital, The Key Laboratory of Zhejiang Province for Aptamers and Theranostics, Hangzhou Institute of Medicine (HIM), Chinese Academy of Sciences, Hangzhou, Zhejiang 310022, China.

[2]Institute of Molecular Medicine, Shanghai Key Laboratory for Nucleic Acid Chemistry and Nanomedicine, Renji Hospital, School of Medicine, Shanghai Jiao Tong University, Shanghai 200127, China.

[3]School of Pharmacy, Shandong Technology Innovation Center of Molecular Targeting and Intelligent Diagnosis and Treatment, Binzhou Medical University, Yantai, 264003, China.

[4]Intellinosis Biotech Co., Ltd., Shanghai, 201112, China.

[5]These authors contributed equally: Linlin Yang, Qian Tang.

*e-mail: dahan@sjtu.edu.cn (D.H.), chaozhang@sjtu.edu.cn (C.Z.), guopei@ibmc.ac.cn (P.G.)

Table of Contents

S1 Data and analysis

Supplementary Figures 1-21……………………………………………………………………3-28

Supplementary Note 1…………………………………………………………………………..11-13

Supplementary Note 2…………………………………………………………………………..19

S2 Synthetic samples and clinical sample information

Supplementary Table 1……………………………………………………………………………29

Supplementary Table 2……………………………………………………………………………30

S3 DNA molecular computation systems comparison

Supplementary Note 3…………………………………………………………………………..31

Supplementary Table 3……………………………………………………………………………31

S4 Sequences of staples and the reaction probes

Supplementary Note 4…………………………………………………………………………..32

Supplementary Table 4…………………………………………………………………………..32-34

Supplementary Note 5…………………………………………………………………………..35

Supplementary Table 5…………………………………………………………………………..35-37

S5 Reaction conditions

Supplementary Table 6……………………………………………………………………………38-39

References……………………………………………………………………………………………40

S1 Data and analysis.

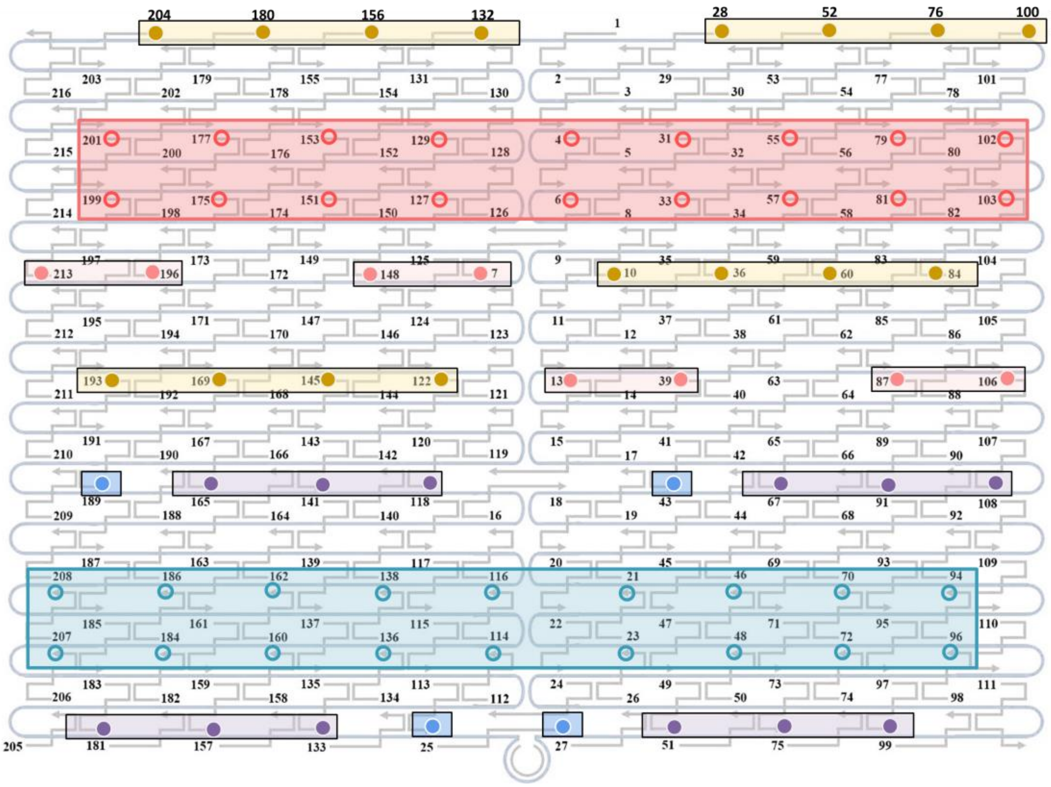

Supplementary Fig. 1 Schematic illustration for the docking sites of DNA framework of DNA IC-CLA with extended staples for anchoring computing probes and reporting probes. The DNA origami structure features four distinct computing zones, each designed to recognize a specific input. Each zone is replicated four times, ensuring that there are four identical copies of each zone on the origami. These zones are equipped with four corresponding computing probes (L_1-A, L_2-B, L_3-C, and L_4-D) that are responsible for sensing inputs (a, b, c, and d) and performing the necessary weighting operations. Additionally, there are two separate reporting zones, each containing 18 docking sites for the immobilization of two distinct reporting probes (RE and RF probes), which are used for output reporting.

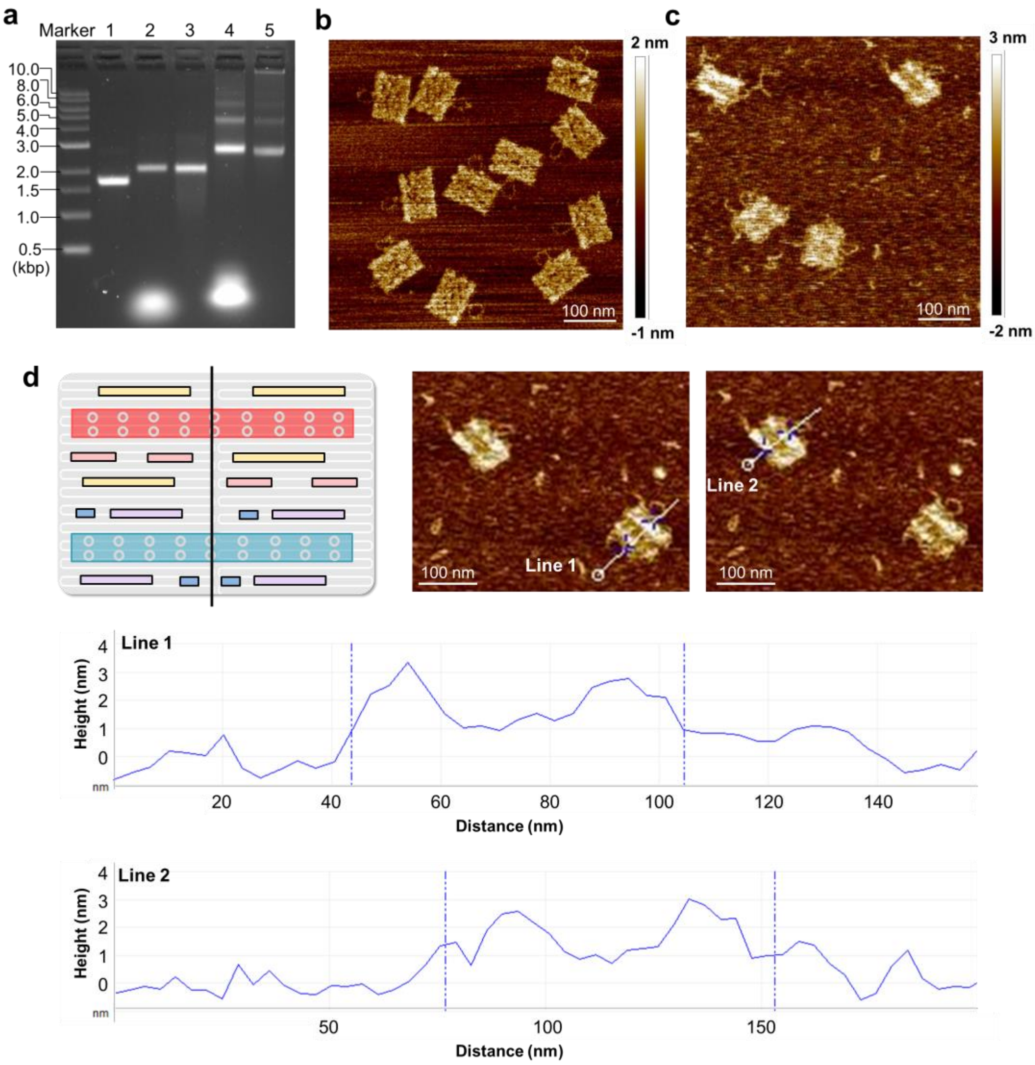

Supplementary Fig. 2 Characterization of DNA IC-CLA. **a**, 1% agarose gel characterization of DNA origami framework and DNA IC-CLA. Lane 1: scaffold; Lane 2: unpurified DNA origami framework; lane 3: purified DNA origami framework; lane 4: unpurified DNA IC-CLA; lane 5: purified DNA IC-CLA. The DNA origamis and DNA IC-CLA were purified by size exclusion chromatography using Sepharose CL-4B. The agarose gel analysis demonstrated that almost all excess staples and DNA probes were removed after purification. **b-c**, The representative gas-phase AFM images of DNA origami framework (**b**) and DNA IC-CLA (**c**). The experiments for **b** and **c** were repeated three times with similar results. **d**, Scheme and the height analysis of gas-phase AFM images of DNA IC-CLA. The height of the blank zone in AFM images of DNA IC-CLA is about 1 nm, and the height of the modified zones in AFM images of DNA IC-CLA is about 3 nm. The uncropped gel in a is shown in Supplementary Fig. 22. Source data are provided as a Source Data file.

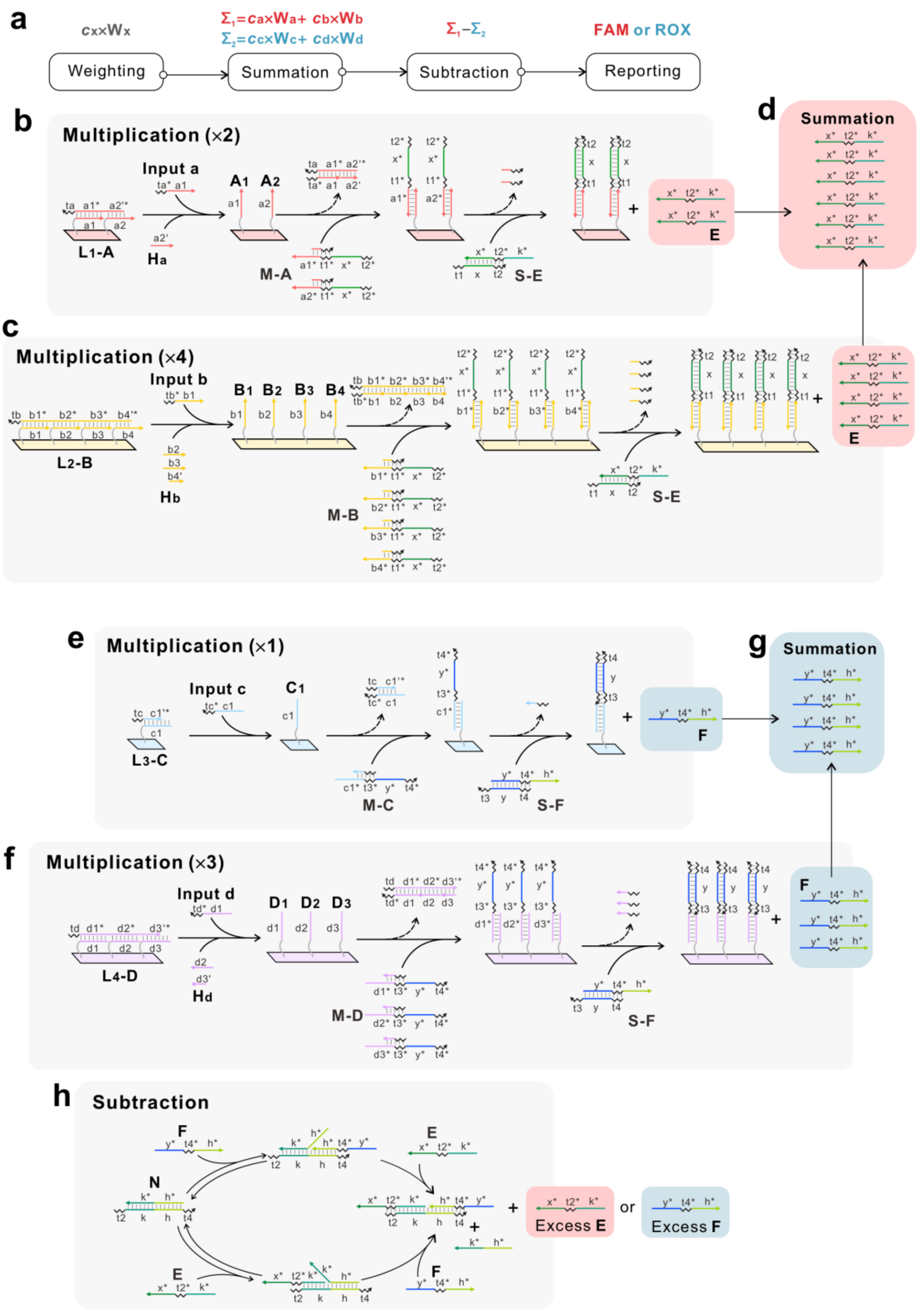

Supplementary Fig. 3 Scheme for DNA computation of DNA IC-CLA. **a**, Workflow of DNA computation of DNA IC-CLA. Scheme for the multiplication of $c(\text{output})_a=2\times c(\text{input})_a$ **(b)**, $c(\text{output})_b=4\times c(\text{input})_b$ **(c)**, $c(\text{output})_c=1\times c(\text{input})_c$ **(e)** and $c(\text{output})_d=3\times c(\text{input})_d$ **(f)** on DNA IC-CLA. Scheme for summation of a×2+b×4= E **(d)** and c×1+d×3= F **(g)** of DNA IC-CLA. **h,** Scheme for subtraction of E – F. This operation is achieved by using an N probe that can annihilate the sums of different types of input.

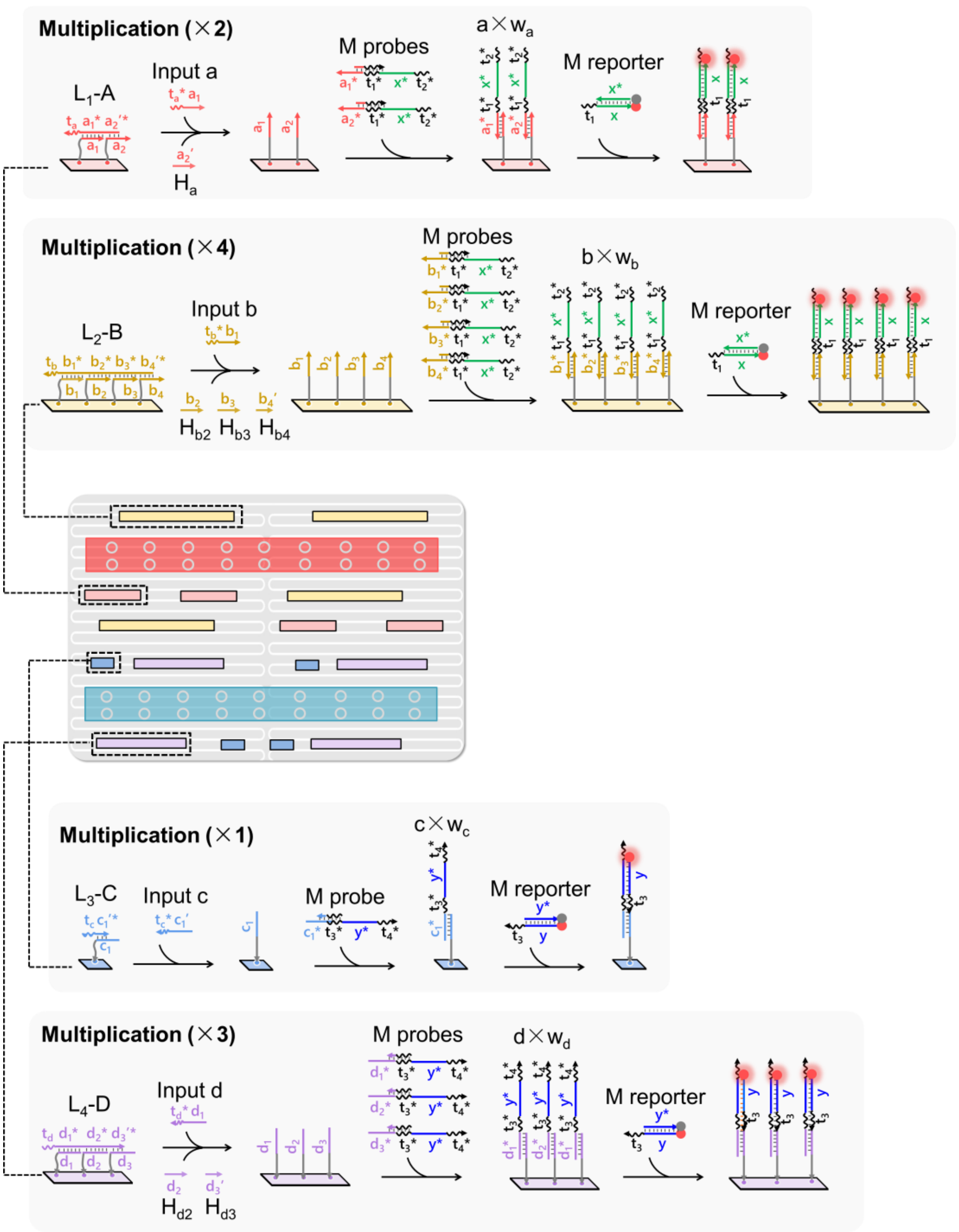

Supplementary Fig. 4 Schematic illustration for the multiplication of DNA IC-CLA. A fluorescent reporting scheme with M reporter is used to confirm the multiplication results of $c(\text{output})_n = w_n \times c(\text{input})_n$. There are four different computing zones for specifically recognizing four different inputs, and each zone is replicated with four identical copies on the origami. The four different computing zones were anchored with four computing probes (L_1-A, L_2-B, L_3-C and L_4-D) for recognizing four inputs (Input a, b, c and d) and executing the defined weighting operations, respectively.

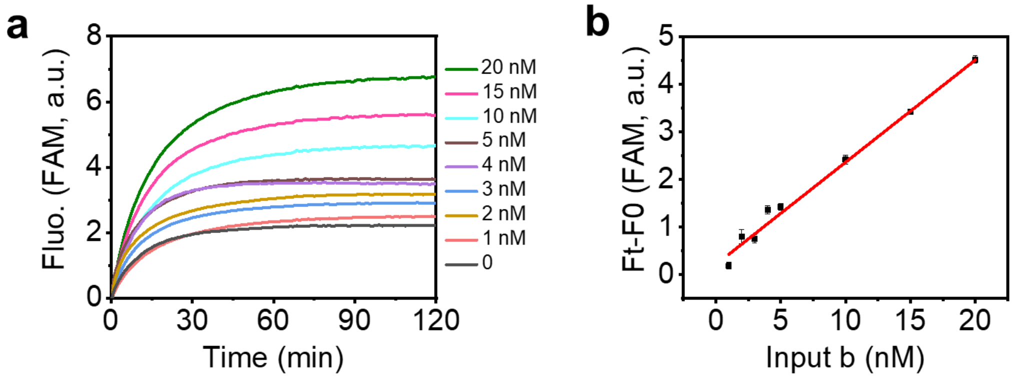

Supplementary Fig. 5 Multiplication results for different concentrations of Input b. **a,** Multiplication fluorescence kinetics of DNA IC-CLA with different concentrations of Input b. **b,** Linear relationships is obtained between Input b concentrations and the steady-state fluorescence response. Fluorescence difference (Ft - F0) at 60 min was used to compare the fluorescence response signals. Ft: steady state fluorescence of the sample. F0: the background fluorescence. Multiplication reactions were carried out with 5 nM of DNA IC-CLA, 25 nM of each H probe, 25 nM of each M probe, and 200 nM of each M reporter and different concentrations of Input b. Data are presented as mean values +/- SD, *n* = 3 biological replicates. Source data are provided as a Source Data file.

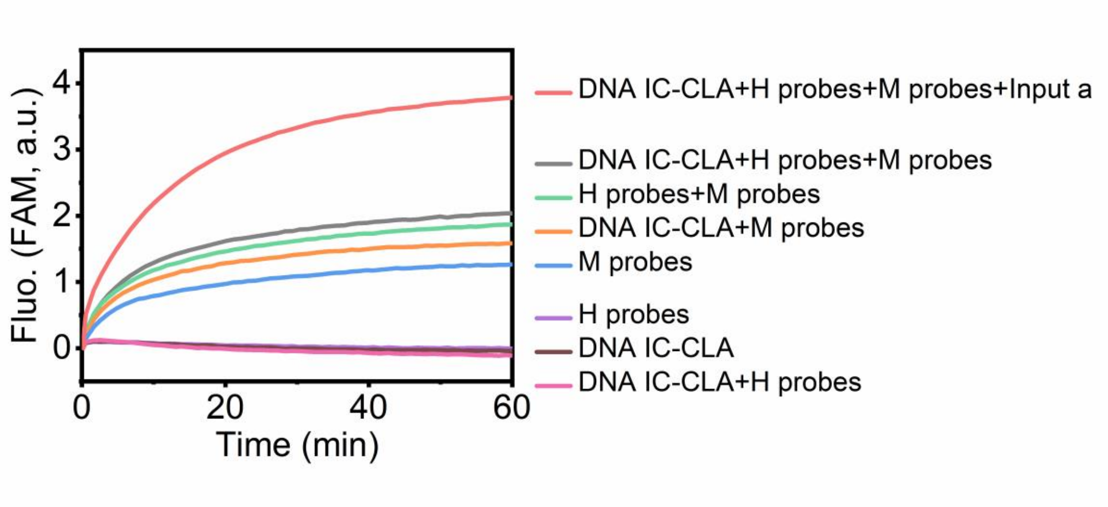

Supplementary Fig. 6 Verification of the leakage source in multiplication operation by multiplication fluorescence kinetics. Multiplication fluorescence kinetics were carried out with 200 nM of M reporter and different combinations of 5 nM of DNA IC-CLA, 25 nM of each H probe, 25 nM of each M probe, 20 nM of Input a. Source data are provided as a Source Data file.

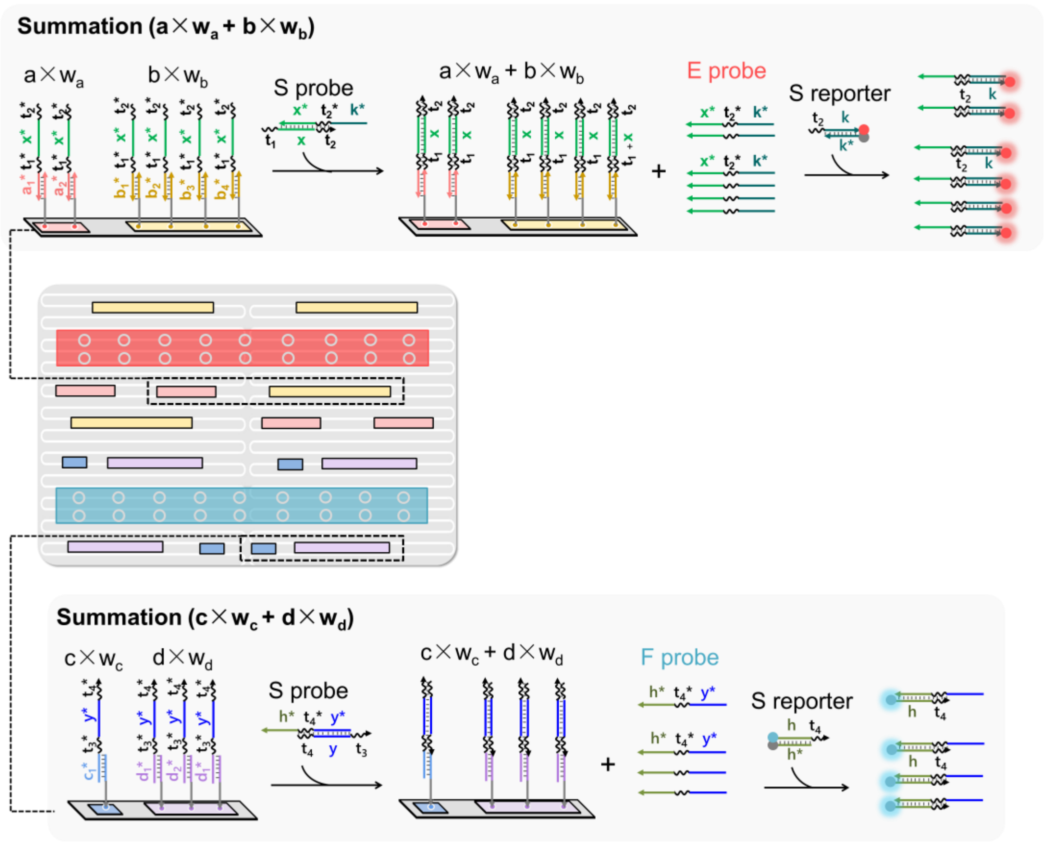

Supplementary Fig. 7 Schematic illustration for the summation of DNA IC-CLA. A fluorescent reporting scheme with S reporter is used to confirm the summation results of a×w_a+b×w_b=E; c×w_c+d×w_d=F. For the summation calculation, we utilized the output (w_n× c(input)$_n$) obtained from multiplications as the input for this step. The weighted outputs (a×w_a, b×w_b, c×w_c and d×w_d) on the origami from the last step are recognized by excess freely diffusible S probes and summed up by forming the complex on the surface of DNA IC-CLA for the implementation of a×w_a+b×w_b and c×w_c+d×w_d.

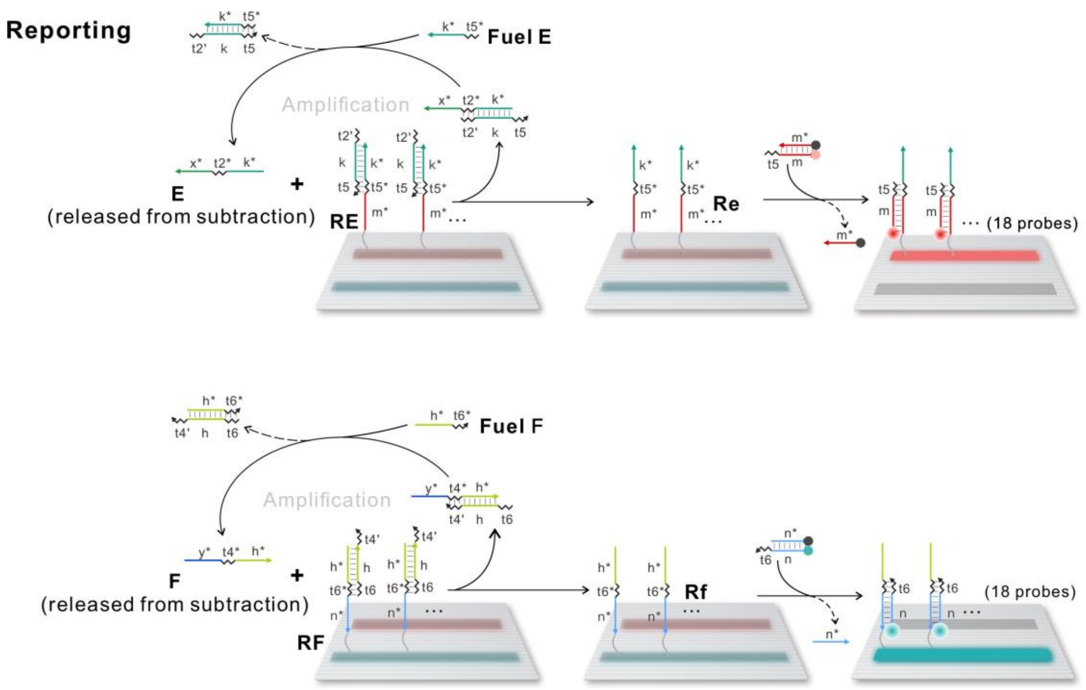

Supplementary Fig. 8 Schematic illustration for entropy-driven catalytic amplification and reporting process of DNA IC-CLA. There are two different reporting zones that contain 18 docking sites for the immobilization of two reporting probes (RE and RF probe) and output reporting on the surface of DNA IC-CLA, respectively. Two distinct fluorescent reporters with FAM and ROX fluorophores were used to report the corresponding outputs on the reporting zones, which were associated with positive and negative weights after the subtraction operation. An entropy-driven catalytic amplification restores the offset signals and improves the reporting sensitivity.

Supplementary Note 1

Simulations

To illustrate the acceleration of computation by localizing computing probes on our DNA-IC CLA, we have performed a simulation to show a comparison of computation efficiency between non-localized and localized conditions. All kinetic simulations were conducted with Python's SciPy package (version 1.2.1) using the solve_ivp() function.

In the simulation model, we assume that most of the DNA strand displacement reactions are reversible. We consider that branch migration is fast so that the reaction rate will mostly depend on toehold binding and subsequent toehold unbinding, which are represented as a simple bimolecular reaction. Since the reaction rate does not primarily depend on sequence length or base composition for 10-100 nt reactants, we assume that the hybridization rate (k_f) of two single strands of DNA with complementary sequences is close to 3×10^{-3} $nM^{-1}s^{-1}$. The dissociation rate (k_r) can be determined using $\Delta G^{o} = -RT\ln K$, where $K = \frac{k_f}{k_r}$. Moreover, we set a factor L which denotes the local concentration and captures the effect of localization.

In multiplication step, to simplify the reaction of signal initiation, all the probes that located on the computing platform are considered as a whole reactant, and helpers that take part in the same multiplication reaction are treated as a reactant as well. Therefore, the reaction of signal initiation can be described as follows:

$$\text{Input} + \text{Helper} + \text{Multiplication\_Platform} \xrightarrow{k1} w \bullet \text{Weight} + \text{waste1} \quad \text{(E.q. 1)}$$

where w represents the weight we gave to the input.

The following pivotal reactions in multiplication can be described as follows:

$$\text{Weight} + \text{M probe} \underset{L\times k3}{\overset{L\times k2}{\rightleftharpoons}} \text{actW} + \text{waste2} \quad \text{(E.q. 2)}$$

Since this local concentration is difficult to compute in the general case[1], we obtained the value of L by fitting the experimental data and found L=1. For summation step, we combined two different multiplication steps with different values of w and make a summation implementation.

$$\text{actW} + \text{S probe} \underset{k5}{\overset{k4}{\rightleftharpoons}} \text{actS} + \text{E probe} \quad \text{(E.q. 3)}$$

We then used I to represent Input, H to represent Helper, W to represent Weight, $actW$ to represent activated Weight, $actS$ to represent activated S probe and O represent the platform. We designed one platform contains 4 repeated functional regions, and therefore we can obtain:

$$\frac{\partial [W]}{\partial t} = k_1 \times [I] \times [H] \times (4 \times w \times [O]) - L \times k_2 \times \frac{[W]}{4 \times w} \times \frac{[M]}{w} + L \times k_3 \times \frac{[actW]}{4 \times w} \times \frac{[W2]}{w}$$

The five rate constants used in multiplication model are listed below:

Rate constant in multiplication model	Value ($nM^{-1}s^{-1}$)
k_1	5×10^{-4}
k_2	5×10^{-4}
k_3	3×10^{-11}
k_4	5×10^{-4}
k_5	3×10^{-11}

The resulting reactions for the subtraction step are as follows: First, E and F probe would bind to the N probe and start a winner-take-all reaction, resulting in excess E or F probes interact with RE probe or RF probe. The rate constants of these reversible interactions that occur via the toehold can be estimated based on toehold length[2]. Then a Fuel strand (Fuel E or Fuel F) can bind to the exposed domain of the waste and restore E probe (or F probe). The resulting free E (or F) probe can then undergo a localized-like reaction, in which the restored E (or F) probe interacts with the most adjacent RE (or RF) probe localized on the platform. To simplify the reaction among E probe, F probe and N probe, we assumed them as a one-step reaction. The reactions in subtraction model can be described as follows:

$$\text{N probe} + \text{E probe} + \text{F probe} \underset{k7}{\overset{k6}{\rightleftharpoons}} \text{NEF} + \text{waste3} \qquad \text{(E.q. 4)}$$

$$\text{E probe} + \text{RE probe} \underset{k9}{\overset{L\times k8}{\rightleftharpoons}} \text{wasteE} + \text{Re probe} \qquad \text{(E.q. 5)}$$

$$\text{wasteE} + \text{Fuel E} \underset{k11}{\overset{k10}{\rightleftharpoons}} \text{E probe} + \text{waste4} \qquad \text{(E.q. 6)}$$

$$\text{F probe} + \text{RF probe} \underset{k13}{\overset{L\times k12}{\rightleftharpoons}} \text{wasteF} + \text{Rf probe} \qquad \text{(E.q. 7)}$$

$$\text{wasteF} + \text{Fuel F} \underset{k15}{\overset{k14}{\rightleftharpoons}} \text{F probe} + \text{waste5}. \qquad \text{(E.q. 8)}$$

The rate constants used in subtraction model are listed as follows:

Rate constants in subtraction model	Value ($nM^{-1}s^{-1}$)
k_6	5×10^{-4}
k_7	5×10^{-11}
k_8	1×10^{-5}
k_9	5.55×10^{-15}
k_{10}	5×10^{-4}
k_{11}	5.97×10^{-16}
k_{12}	1×10^{-5}
k_{13}	8.19×10^{-15}
k_{14}	5×10^{-4}
k_{15}	3.74×10^{-14}

Additionally, we determined the value of *L* through a fit to the experimental data (Supplementary Figures 9 and 10c). This analysis revealed that the interaction rate between E (or F) probe and RE (or RF) probe is *L* (*L*=10) times more rapid when localized compared to non-localized conditions. In our simulations, we utilized the experimental concentrations of RE (or RF) probes under both non-localized and localized conditions, with the sole variable being *L*, which was set to 1 for non-localized and 10 for localized conditions. Ultimately, a Reporter complex can bind to the exposed domain of the opened RE (or RF) probe (designated as Re or Rf probe) to elicit a response.

In our simulations, we observed that the model is sensitive to the rate constants of E (or F) binding to RE (or RF) as well as the restoration of E and F probes. This suggests that localization could significantly influence the catalytic step.

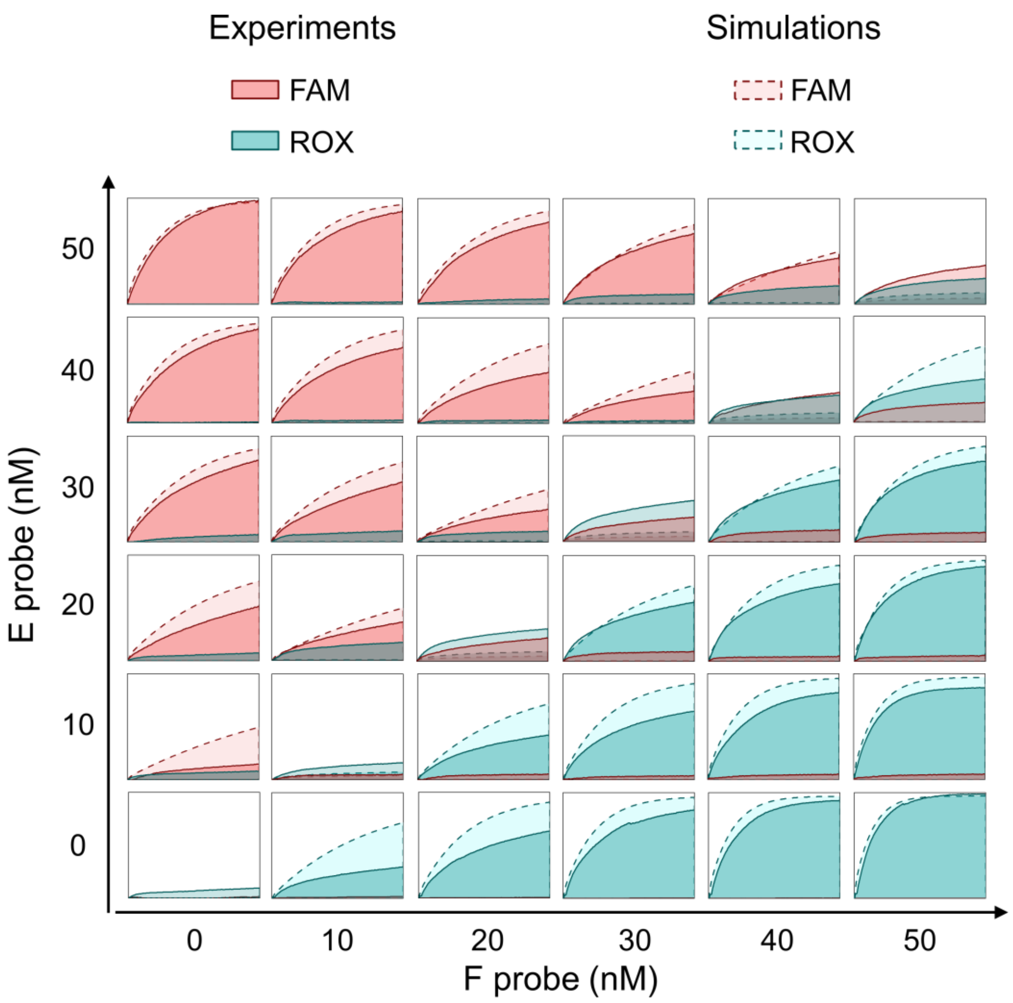

Supplementary Fig. 9 The experimental fluorescence data (solid lines) and the simulation data (dashed lines) of subtraction kinetics of DNA IC-CLA with different concentrations of E probe and F probe. The *x* axis of each small graph is time (from 0–120 min), and the *y* axis is the normalized signal. Subtraction reactions were carried out with 5 nM of DNA IC-CLA, 200 nM of N probe, 200 nM of each Fuel probe (Fuel E and Fuel F), 200 nM of each reporter and different concentrations of E probe and F probe. Source data are provided as a Source Data file.

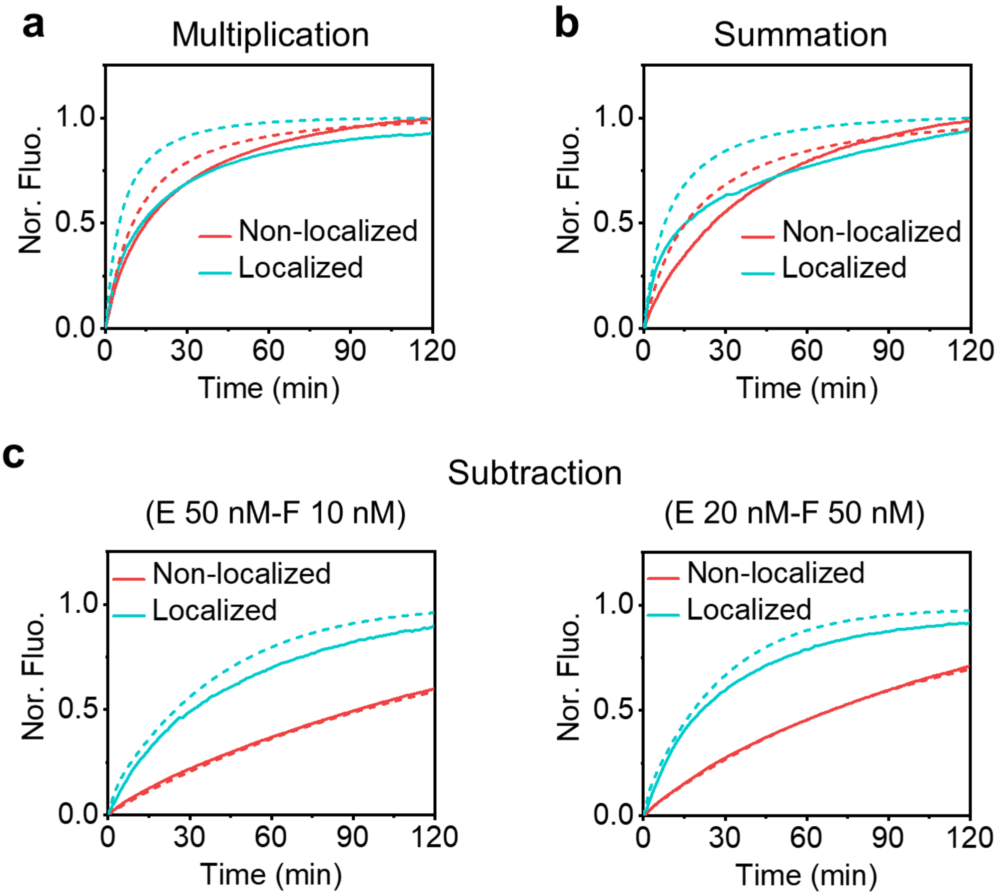

Supplementary Fig. 10 Fluorescence kinetics of multiplication **(a)**, summation **(b)**, and subtraction **(c)** of non-localized system and DNA IC-CLA. Solid lines represent the data from experiment, and dashed lines come from the simulation. Multiplication reactions were carried out with 5 nM of DNA IC-CLA or 20 nM of each Ln-N, 25 nM of each H probe, 25 nM of each M probe, 200 nM of each M reporter, and 20 nM of Input a. Summation reactions were carried out with 5 nM of DNA IC-CLA or 20 nM of each Ln-N, 25 nM of each H probe, 25 nM of each M probe, 150 nM of each S probe, 200 nM of each S reporter, and 20 nM of Input a and Input b. Subtraction used 50 nM of E probe with 10 nM of F probe (left) and 20 nM of E probe with 50 nM F probe (right) as a comparison example. Subtraction reactions were carried out with 5 nM of DNA IC-CLA or 20 nM of each L_n-N, 200 nM of N probe, 200 nM of each Fuel probe (Fuel E and Fuel F), 200 nM of each reporter and different concentrations of E probe and F probe. Source data are provided as a Source Data file.

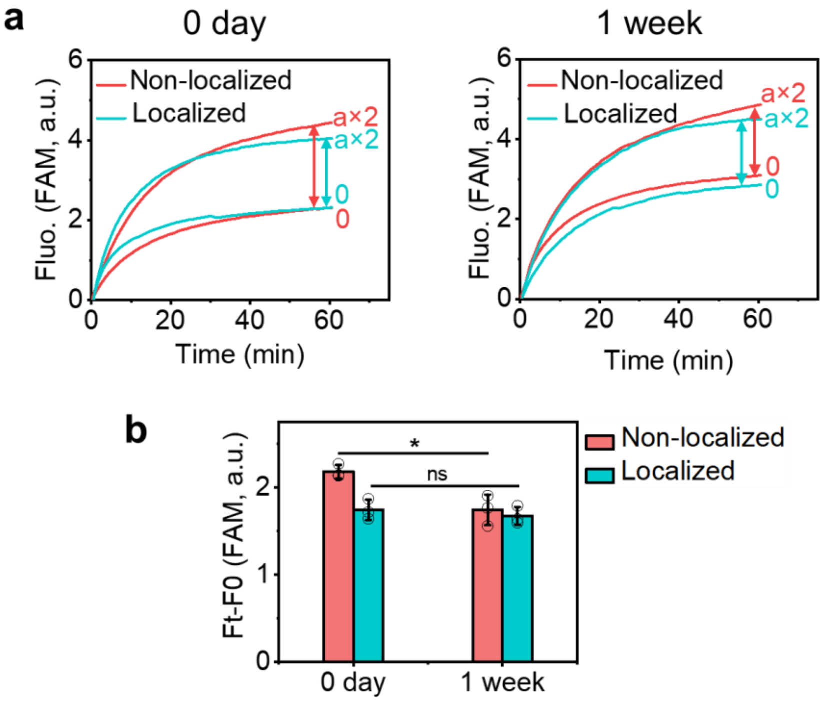

Supplementary Fig. 11 Multiplication fluorescence kinetics **(a)** and fluorescence difference (Ft - F0) **(b)** of non-localized system and DNA IC-CLA in response to Input a, which are freshly prepared and prepared for 1 week, respectively. Multiplication reactions were carried out with 5 nM of DNA IC-CLA or 20 nM of each L_n-N, 25 nM of each H probe, 25 nM of each M probe, 200 nM of each M reporter, and 20 nM of Input a. Data are presented as mean values +/- SD, n = 3 biological replicates. Statistical analysis by two-tailed unequal variance t-test, *$P < 0.05$. Exact p-values are provided in the Source Data file. Source data are provided as a Source Data file.

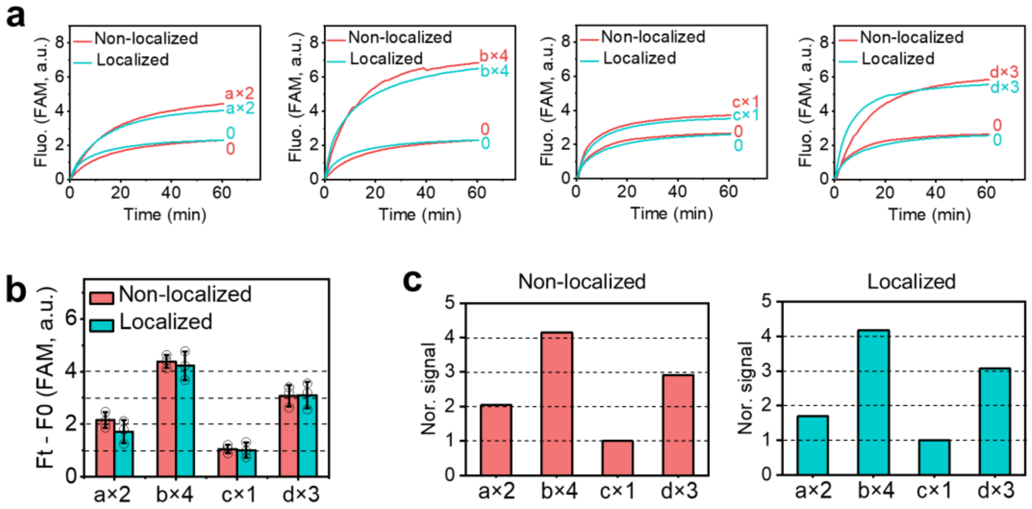

Supplementary Fig. 12 Weighting operations of non-localized system and DNA IC-CLA. **a,** Multiplication fluorescence kinetics of non-localized system and DNA IC-CLA in response to Input a, b, c and d with weights of 2, 4, 1 and 3, respectively. **b,** Fluorescence difference (Ft - F0) of non-localized system and DNA IC-CLA in response to Input a, b, c and d with weights of 2, 4, 1 and 3, respectively. Data are presented as mean values +/- SD, n = 3 biological replicates. **c,** Normalized fluorescence signals were used to compare the conformity of non-localized system and DNA IC-CLA with the pre-defined weights of 1, 2, 3 and 4, respectively. The calibration system stipulates the fluorescence signal of the sample with the addition of Input c to 1 to calibrate the fluorescence signals of the remaining three samples. Multiplication reactions were carried out with 5 nM of DNA IC-CLA or 20 nM of each L_n-N, 25 nM of each H probe, 25 nM of each M probe and 200 nM of each M reporter, and 20 nM of Input a, b, c and d, respectively. Source data are provided as a Source Data file.

a

Sample	1	2	3	4	5	6	7	8
Input a (nM)	20.0	0.0	0.0	0.0	21.6	10.9	0.4	0.2
Input b (nM)	0.0	20.0	0.0	0.0	18.4	13.7	0.2	0.2
Input c (nM)	0.0	0.0	20.0	0.0	1.1	0.8	9.8	9.0
Input d (nM)	0.0	0.0	0.0	20.0	1.3	0.3	15.3	14.9
f(x)	40.0	80.0	-20.0	-60.0	111.8	74.9	-54.1	-52.5

b

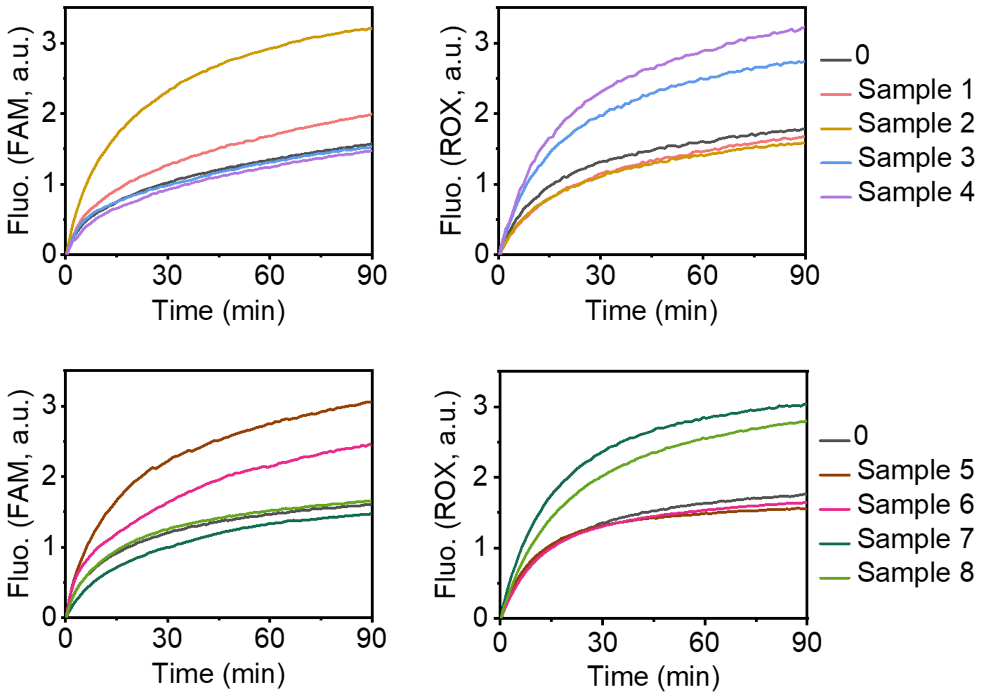

Supplementary Fig. 13 Classification of DNA IC-CLA for varying combinations of DNA inputs. **a,** Combinations information of DNA inputs of 8 samples. **b,** Fluorescence kinetics of DNA IC-CLA for 8 samples in response to different combinations of DNA input concentrations (No. 1-8). The entire computations were carried out with 5 nM of DNA IC-CLA, 25 nM of each H probes, 25 nM of each M probe, 150 nM of each S probe, 200 nM of N probe, 200 nM of each Fuel probe (Fuel E and Fuel F), 200 nM of each reporter, and different combinations of DNA input concentrations. Source data are provided as a Source Data file.

Supplementary Note 2

Classification margin of DNA-IC CLA

Our analysis began by examining the separation margin of the subtraction operation within the DNA IC-CLA, focusing on various combinations of weighted sums (specifically, E and F probes). The concentration range tested for E and F probes spanned from 0 to 50 nM. In Supplementary Figure 14a, the upper-left, diagonal, and lower-right areas correspond to the results for E<E, E=F, and E>F, respectively. We observed that signal ambiguity arose when the concentrations of the two weighted sums were too similar, leading to the misclassification of 7 samples (indicated by grey dots). We have designated the area where E-F = ± 10 nM as the separation margin for subtraction, within which 19 samples were challenging to classify accurately. The remaining 30 samples that fell beyond this margin produced the expected results. In summary, the findings indicate that DNA-IC CLA can reliably differentiate samples when the absolute difference between E and F exceeds 10 nM.

Building upon these results, we prepared 40 samples with $f(x)$ values approximately centered around 10 nM to validate the experimental classification margin for the function $f(x) = 2\times c(\mathrm{a}) + 4\times c(\mathrm{b}) - 1\times c(\mathrm{c}) - 3\times c(\mathrm{d})$ (Supplementary Figure 14b). The results indicated that 20 samples with $f(x)$ values within the range of $f(x) = \pm$ 10 nM (marked by grey lines) were difficult to classify correctly, including 16 samples that were misclassified (grey dots). The remaining 20 samples, which were well beyond this margin, were accurately reported. When analyzing synthetic and clinical samples, we employ LATE-PCR to amplify the target sequences, ensuring that the values of $f(x)$ remain within a classifiable range.

We have evaluated the classification performance of our in silico-trained classifier using a validation set comprising 270 non-small cell lung cancer (NSCLC) and 27 healthy samples from the TCGA database (Supplementary Figure 14c). The classifier output values for 17 samples (5.7%) fall within the range of ± 10 (indicated by grey lines), which presents a challenge for accurate classification. Among these, 5 samples (1.7%, represented by grey dots) lie on the incorrect side of the separation line (dotted line) and are deemed impossible to classify correctly. The remaining 280 samples (94.3%) are well outside this margin, suggesting that our classifier is expected to achieve high diagnostic accuracy for NSCLC. The small proportion of samples within the separation margin indicates that our classification model is robust and suitable for high-precision NSCLC diagnosis.

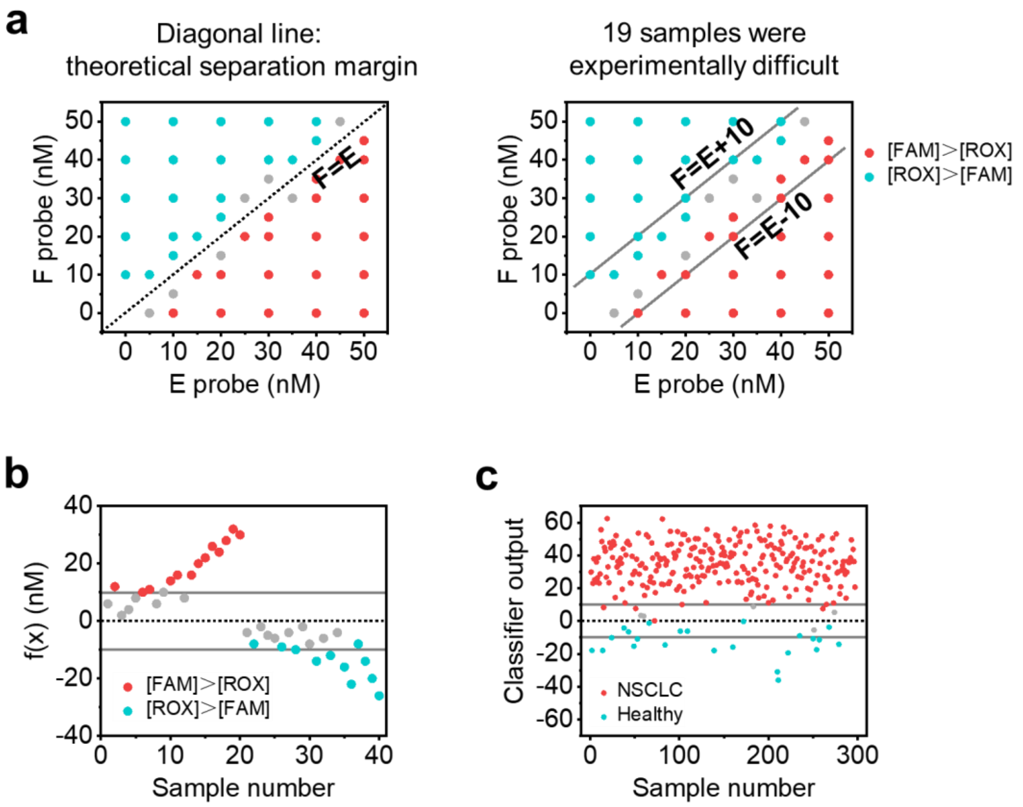

Supplementary Fig. 14 Classification margin of DNA-IC CLA. **a,** Fluorescence readings of DNA IC-CLA for samples with varying concentrations of E probe and F probe (grey dots represent incorrectly reported samples). **b,** Performance of DNA IC-CLA for samples with varying *f*(*x*) (grey dots represent incorrectly classified samples). **c,** Performance of the selected classifier model in the training set, where 292 (98.3%) samples were classified correctly. The validation set includes miRNA-seq data from 270 NSCLC and 27 healthy individuals in the TCGA database (https://portal.gdc.cancer.gov)[3]. Source data are provided as a Source Data file.

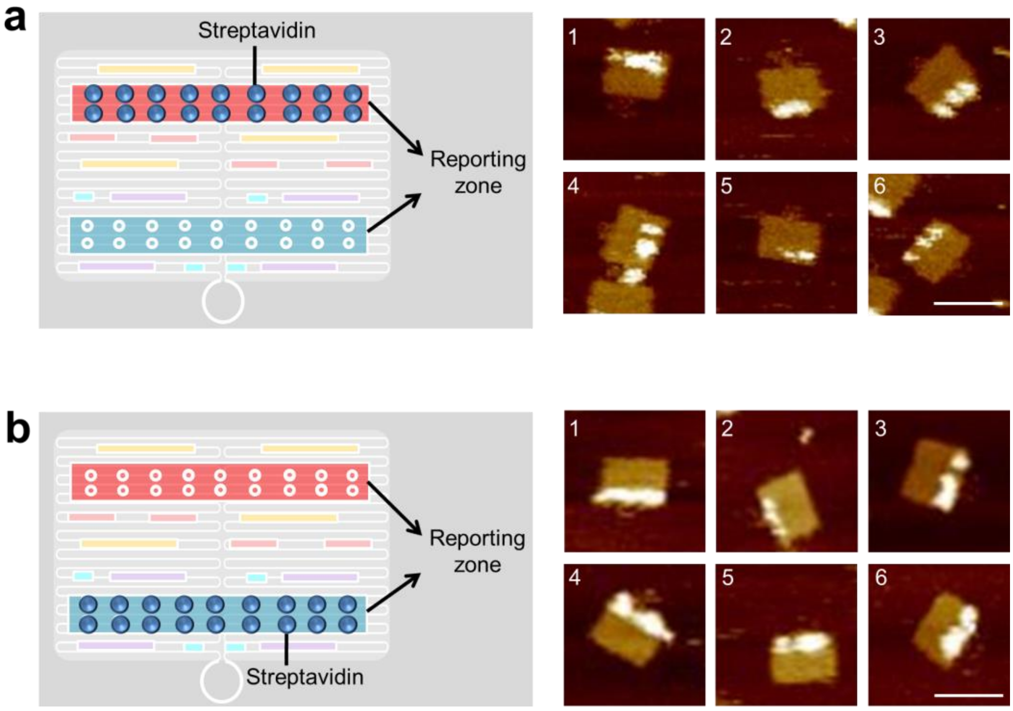

Supplementary Fig. 15 The representative AFM images of individual DNA IC-CLA in response to Input a **(a)** or Input c **(b)**. Scale bar, 100 nm. Two reporting zones can be distinguished by the loop of unfolded part of M13 DNA. The reaction was carried out with 5 nM of DNA IC-CLA, 25 nM of each H probes, 25 nM of each M probe, 150 nM of each S probe, 200 nM of N probe, 200 nM of each Fuel probe (Fuel E and Fuel F), 200 nM of each biotin-labeled reporter, and 20 nM of Input a or Input c. The experiments were repeated by three times with similar results.

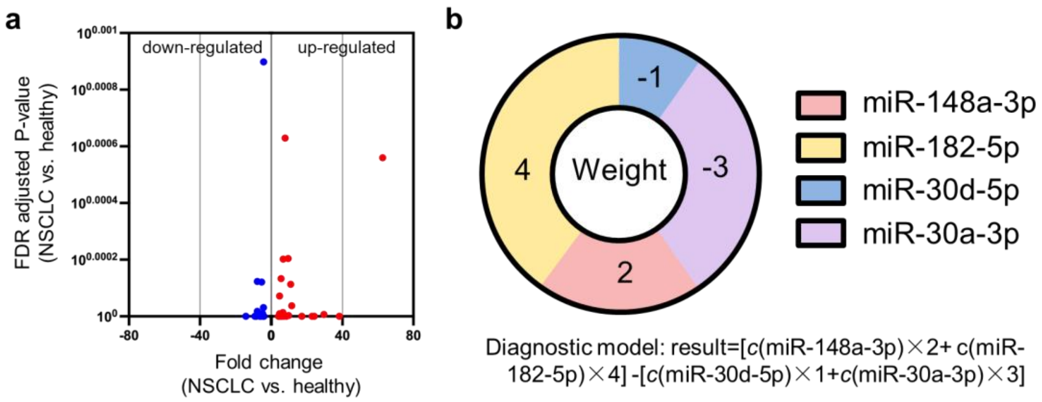

Supplementary Fig. 16 In silico trained SVM classifier for NSCLC diagnosis. **a,** Differential gene analysis of miRNA-seq data from TCGA. FDR means false discovery rate. Fold change is used to calculate the differential multiples of gene expression values between cancer and normal samples. **b,** Selected miRNA combinations and their associated weights for the classifier model. Source data are provided as a Source Data file.

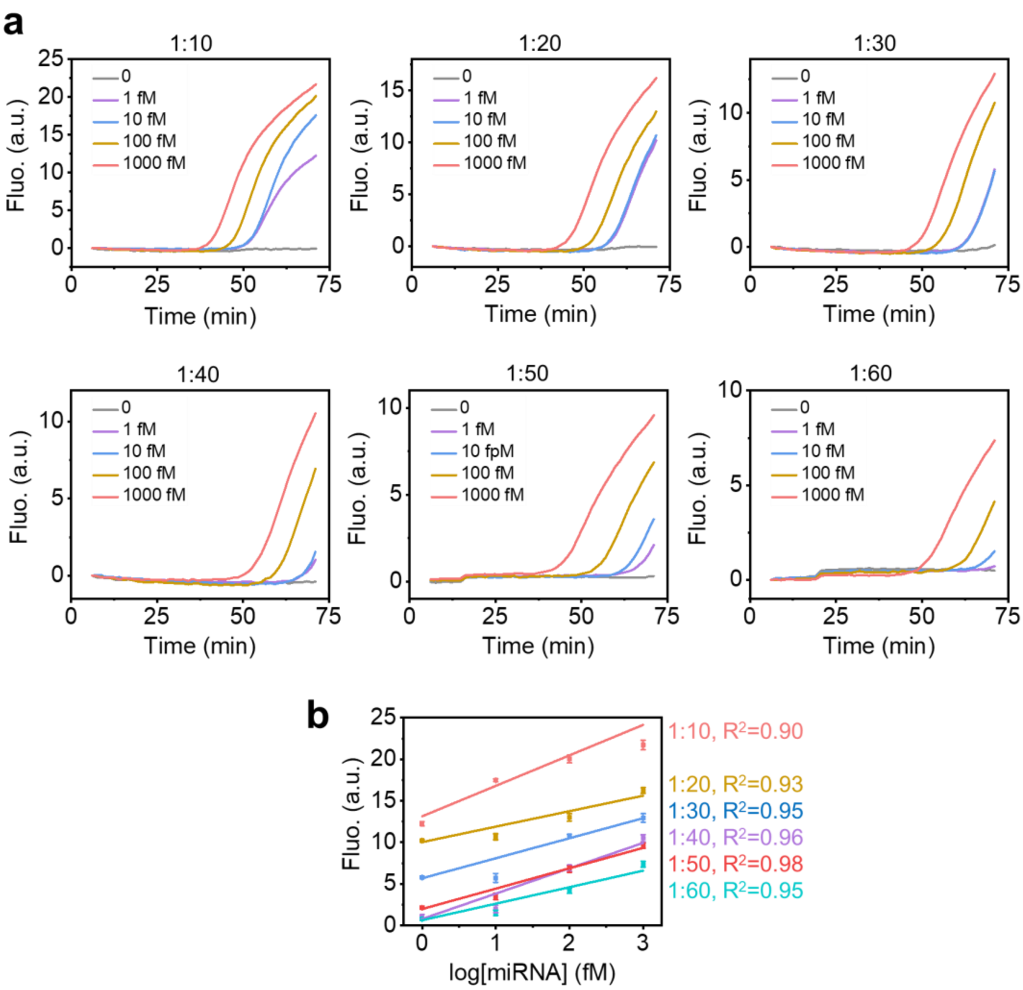

Supplementary Fig. 17 Optimization of forward (F) and reverse primer (R) ratios in LATE-PCR. **a,** Detection of LATE-PCR amplification products of miR-148a-3p with different forward (F) and reverse primer (R) ratios using a FAM-labeled TaqMan probe. **b,** Plot of fluorescence of PCR at cycle 50 versus initial miRNA (miR-148a-3p) concentrations from 1 fM to 1 pM (F:R=1:10, R^2= 0.90; F: R=1:20, R^2= 0.93; F: R=1:30, R^2= 0.95; F: R=1:40, R^2= 0.96; F: R=1:50, R^2= 0.98; F: R=1:60, R^2= 0.95). Data are presented as mean values +/- SD, n = 3 biological replicates. Source data are provided as a Source Data file.

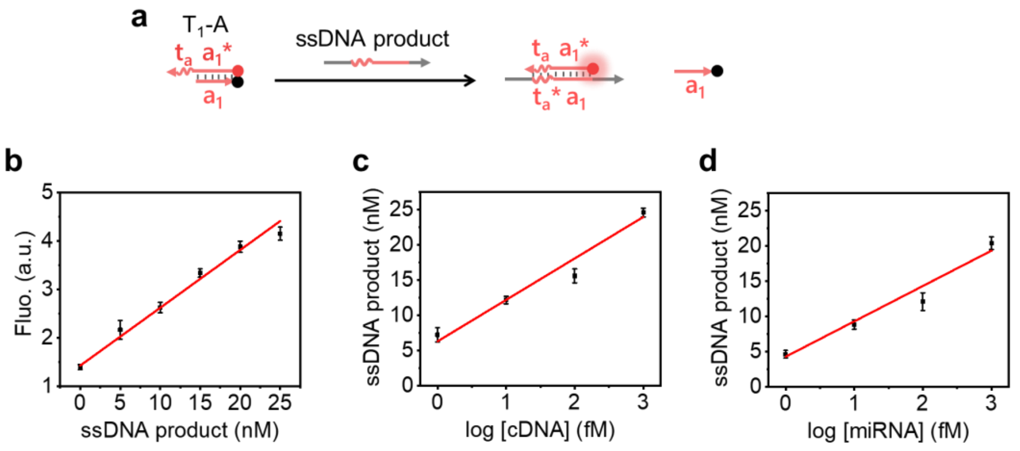

Supplementary Fig. 18 Quantification of ssDNA product post-LATE-PCR. **a,** Scheme of quantification of ssDNA product post-LATE-PCR using T_1-A probe truncated from L_1-A probe. **b,** Synthetic ssDNA with known concentrations vs. fluorescence were quantified by T_1-A probe. **c,** cDNA amplicon after LATE-PCR vs. fluorescence. **d,** miRNA amplicon after LATE-PCR vs. fluorescence. The reactions were carried out with 20 nM of T_1-A probe, and different concentrations of synthetic ssDNA products and LATE-PCR amplification products of synthetic cDNA or miRNA (miR-148a-3p). Data are presented as mean values +/- SD, *n* = 3 biological replicates. Source data are provided as a Source Data file.

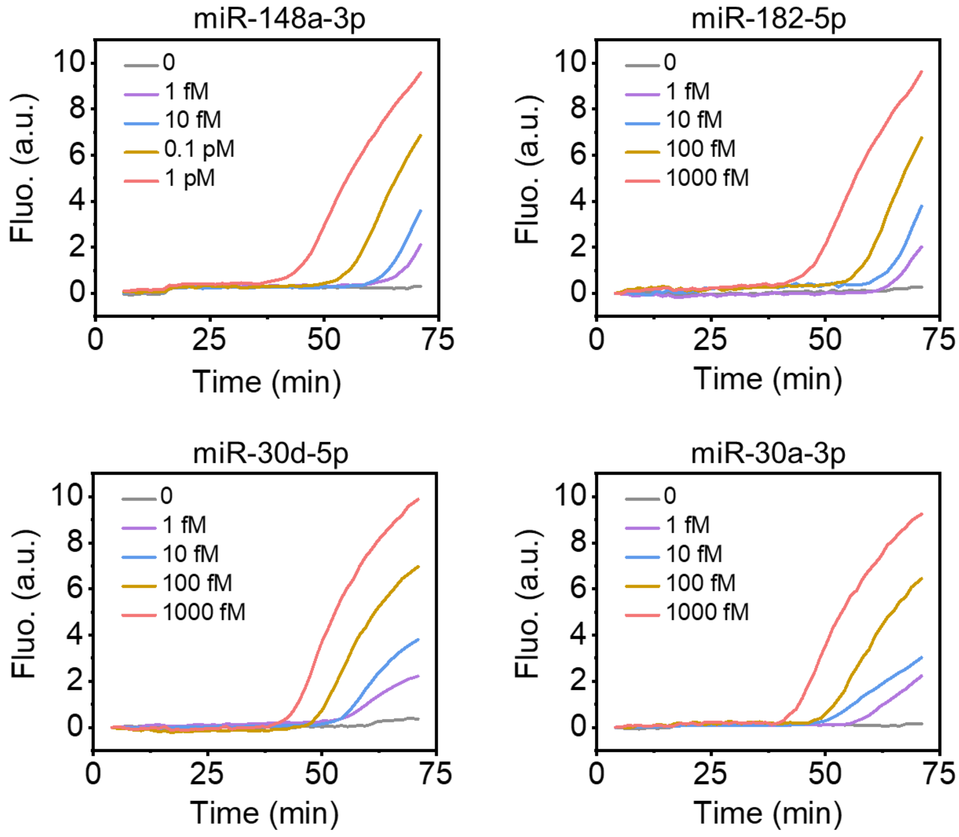

Supplementary Fig. 19 LATE-PCR amplification efficiency for the four selected miRNAs (miR-148a-3p, miR-182-5p, miR-30d-5p, miR-30a-3p). Detection of LATE-PCR amplification products with different initial concentrations of miRNAs using corresponding primers, enzymes and FAM-labeled TaqMan probe. Source data are provided as a Source Data file.

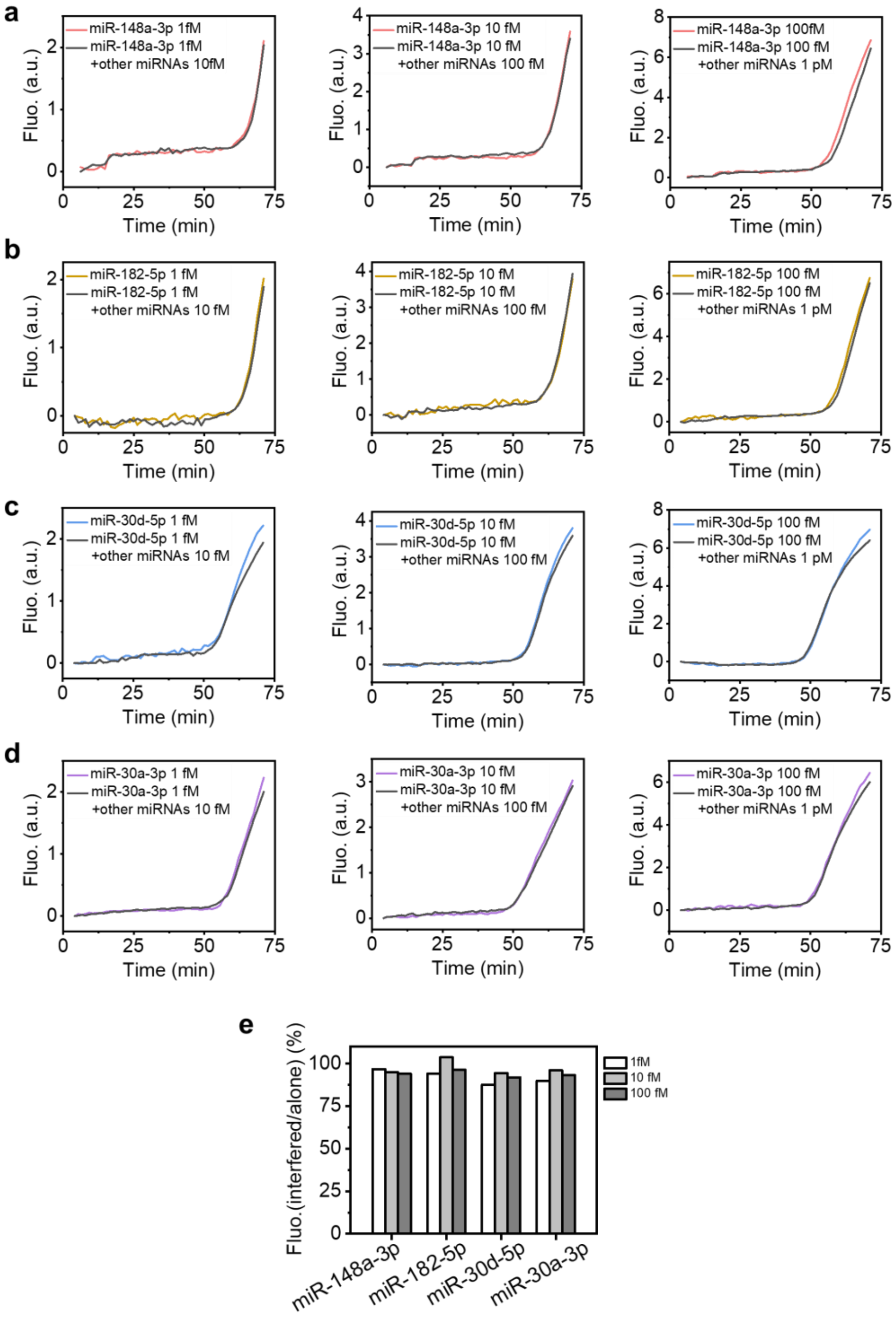

Supplementary Fig. 20 LATE-PCR amplification of the specific miRNA alone and the specific miRNA with 10-fold excess of the other three miRNAs (**a,** miR-148a-3p; **b,** miR-182-5p; **c,** miR-30d-5p; **d,** miR-30a-3p). **e,** Fluorescence ratios of LATE-PCR amplification of the specific miRNA interfered with other miRNAs to miRNA alone. Source data are provided as a Source Data file.

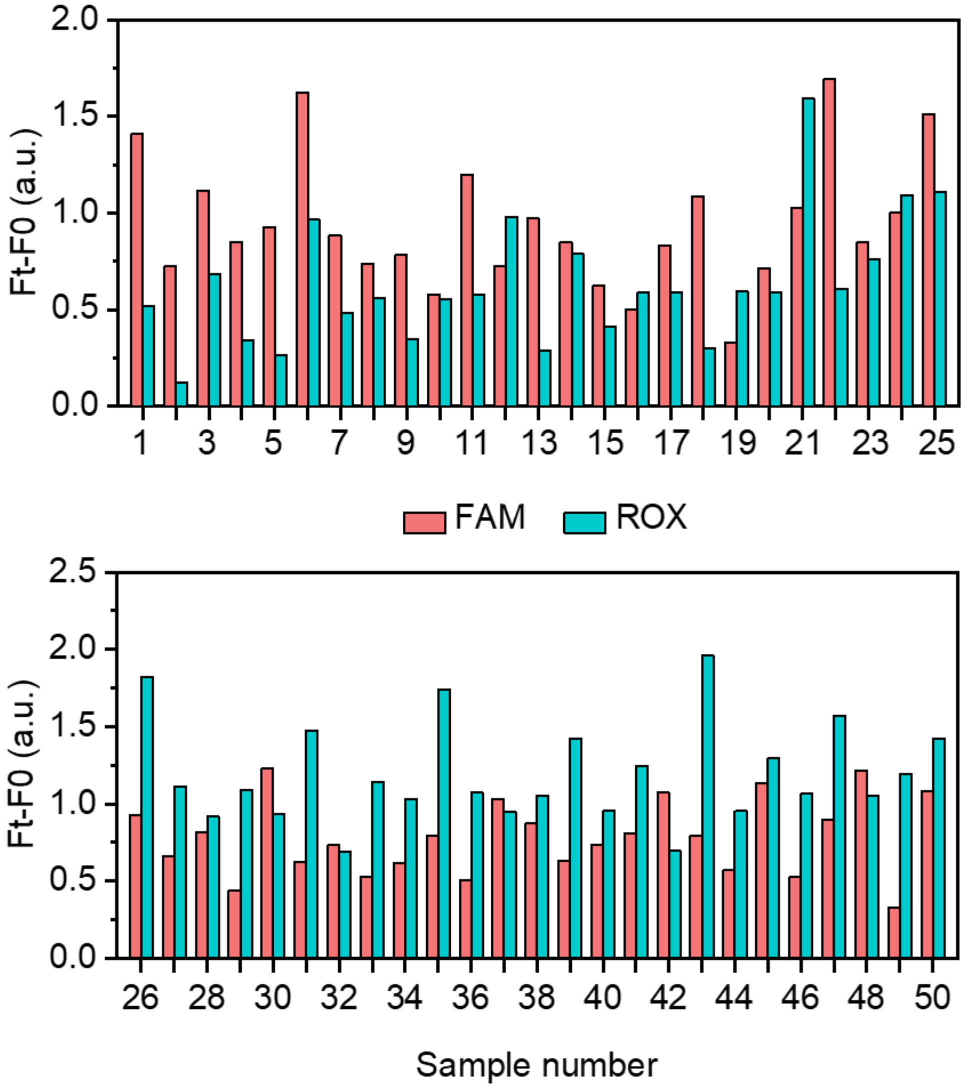

Supplementary Fig. 21 Fluorescence readings of 50 clinical serum samples. Fluorescence differences (Ft - F0) of the clinical NSCLC samples (No. 1-25) and clinical healthy samples (No. 26-50). Ft: steady state fluorescence of the sample. F0: the background fluorescence. [Ft-F0(FAM)] - [Ft-F0(ROX)] > 0.05 belongs to NSCLC, [Ft-F0(ROX)] - [Ft-F0(FAM)] > 0.05 belongs to healthy. Source data are provided as a Source Data file.

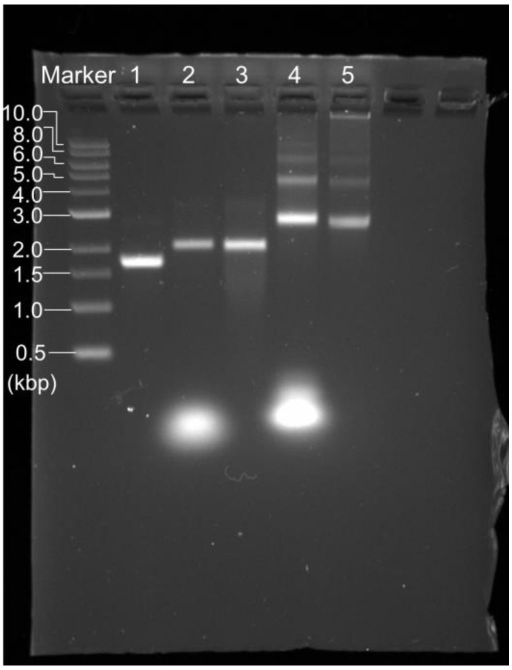

Supplementary Fig. 22 Uncropped gel in Supplementary Fig. 2a.

S2 Synthetic samples and clinical samples information.

Supplementary Table 1. The synthetic samples information.

NSCLC samples: In theory, diagnostic result > 0, [FAM] > [ROX]															
	1	2	3	4	5	6	7	8	9	10	11	12	13	14	15
miR-148a-3p (fM)	107.8	54.4	77.0	69.2	42.2	27.1	75.2	41.7	39.6	47.3	34.5	37.9	82.2	49.3	71.1
miR-182-5p (fM)	91.9	68.4	21.3	11.0	3.0	8.8	49.2	26.5	10.5	5.9	16.2	18.8	18.8	8.8	9.3
miR-30d-5p (fM)	5.3	4.0	4.5	5.5	2.6	4.2	18.3	6.1	7.6	9.1	10.8	7.4	4.3	5.5	20.5
miR-30a-3p (fM)	6.5	1.5	7.5	2.0	1.4	0.3	3.0	2.6	1.3	1.0	1.2	3.0	8.8	4.8	14.7
Healthy samples: In theory, diagnostic result < 0, [FAM] < [ROX]															
	16	17	18	19	20	21	22	23	24	25	26	27	28	29	30
miR-148a-3p (fM)	1.8	0.9	1.7	1.4	2.0	1.0	0.7	0.5	2.5	1.8	2.9	1.2	1.3	2.8	2.7
miR-182-5p (fM)	0.8	0.8	0.9	0.6	1.0	0.4	0.9	0.8	0.8	0.6	0.3	0.8	0.6	0.3	0.8
miR-30d-5p (fM)	49.2	44.8	46.3	48.5	40.9	31.7	41.6	31.1	36.1	35.2	38.7	42.2	38.6	30.4	35.5
miR-30a-3p (fM)	76.3	74.6	76.3	59.3	44.1	67.9	75.1	62.5	50.2	69.7	62.7	42.0	31.6	49.1	58.6

Supplementary Table 2. The summary of the clinical samples information.

Clinical Characteristics	NSCLC					Healthy
Cancer type	Adenocarcinoma			Squamous cell carcinoma		/
Stage (TNM)	IA	IB	IIA	IB	IIA	/
Cases	16	2	1	4	2	25
Age (year) Mean ± SD	62.8±8.9	65.5±0.5	51	66.3±3.6	73.5±1.5	66.8±9.1
Gender Male / Female	4/12	1/1	1/0	3/1	2/0	19/6

S3 DNA molecular computation systems comparison.

Supplementary Note 3

The design and application of DNA molecular computation exhibit considerable variation across studies, which complicates the process of quantitative comparison. Typically, systems that feature fewer cascade layers or employ localized circuits exhibit superior kinetics. Conversely, more extensive systems, with their increased complexity, are more adept at managing sophisticated tasks like pattern recognition[4].

Supplementary Table 3. Comparison between DNA IC-CLA and other DNA molecular computation systems.

References	Scale (numbers of computation steps)	Localized or diffused	Time required for reporting	Applications
Neural network computation with DNA strand displacement cascades[5]	Large	Diffused	8-60 hours	Artificial neurons for a Hopfield associative memory
A spatially localized architecture for fast and modular DNA computing[6]	Small	Localized	2-3 hours	Elementary Logic algorithm
Scaling up molecular pattern recognition with DNA-based winner-take-all neural networks[4]	Large	Diffused	8-20 hours	Pattern recognition
A molecular multi-gene classifier for disease diagnostics[7]	Medium	Diffused	2 hours for computing, 6 hours for classification	Classification of synthetic samples
Cancer diagnosis with DNA molecular computation[8]	Medium	Diffused	6 hours	Diagnosis of synthetic and clinical samples
Nonlinear decision-making with enzymatic neural networks[9]	Small	Diffused	6-16 hours	Non-linear classification
This work	Medium	Localized	3 hours	Diagnosis of synthetic and clinical samples

S4 Sequences of staples and the reaction probes.

Supplementary Note 4

Sequence design of DNA probes

All DNA probes used in the DNA computation have 30–70% GC content and contain short toehold domains and long branch migration domains to prevent secondary structure formation, reduce undesired strand interactions, and ensure desired strand displacement reaction. The a_2' domain (17 nt) of H_a is shorter than a_2 domain (27 nt) of A_2, which increased the effective strand-displacement reaction rate between H_a and L_1-A probe in the presence of Input a, rather than between H_a and M probes. Similarly, H_{b4}, Input c and H_{d3} preferentially react with L_2-B, L_3-C and L_4-D probe, respectively, rather than with M probes, thus reducing leakage. N probe has 8 nt toeholds compared to the 6 nt toeholds in RE or RF probes, which enhances the effective strand-displacement reaction rate between the N probe and E or F probes, compared to that between RE or RF and E or F probes, respectively. This allows E and F probes to execute the subtraction calculation first, followed by a catalytic amplification. Lastly, the candidate sequences were validated using NUPACK for binding energy and specificity assessment. Sequences of the reaction probes are shown below:

Supplementary Table 4. Sequences of the reaction probes.

Name	Sequence and modifications (5′ to 3′)
L_1	CATACTCCAACCTAACACACTCATTCATCGGTAGCTAGTCAACGGTCAACAAA G
A_1	AATGAGATATGGGTAGGGAGTTTTTCGTTGACTAGCTACCGATGAATGAGTG
A_2	AGGTGTGGGTAGGAGTTAGTTTTTTTGTTAGGTTGGAGTATGTTATCAGTGC
Input a	GCGCGTCAGTGCACTACAGAACTTTGTTGACCGTTGACTAGCTACCGACCTCA CTCCTCAGACTC
H_a	TGAATGAGTG TGTTAGGTTGGAGTATG
M probe-A_1 bottom	ATCTATCCACCTCCACCTCTACACCCCACTCATTCATCGGTAGCTAGTCAACG
M probe-A_1 top	AGCTACCGATGAATGAGTGGGGTGT
M probe-$A_{2\text{-}}$ bottom	ATCTATCCACCTCCACCTCTACACCCGCACTGATAACATACTCCAACCTAACA
M probe-A_2 top	TGGAGTATGTTATCAGTGCGGGTGT
L_2	CATACACTTCCACAATCATATCCATTCTATCATACCATCTTCAACCACTATACTT CTAACAATCTCTATCATCTCATCTTTCTGGTGATGGAGCGTGA AGATAGTGTG
B_1	GCGCTACTGGACGCTACCATTTTTTTCACGCTCCATCACCAGAAAGATGAGA
B_2	CCCAACGTGTTTAGAGGAACTTTTTTGATAGAGATTGTTAGAAGTATAGTGG
B_3	TGTCTGAGACCCAAGCAATCTTTTTTTGAAGATGGTATGATAGAATGGATAT
B_4	GAATGTCCGCGTTACCCGTCTTTTTGATTGTGGAAGTGTATGATGAGATGAG
Input b	CGGCCTTGGCAATGGTAGAACTCACACTATCTTCACGCTCCATCACCAGTCCCT AAATCACACGCG
H_{b2}	AAAGATGAGA TGATAGAGATTGTTAGA
H_{b3}	AGTATAGTGG TTGAAGATGGTATGATA
H_{b4}	GAATGGATAT GATTGTGGAAGTGTATG
M probe-B_1 bottom	ATCTATCCACCTCCACCTCTACACCCTCTCATCTTTCTGGTGATGGAGCGTGA
M probe-B_1 top	CATCACCAGAAAGATGAGA GGGTGT

M probe-B_2 bottom	ATCTATCCACCTCCACCTCTACACCCCCACTATACTTCTAACAATCTCTATCA
M probe-B_2 top	ATTGTTAGAAGTATAGTGGGGGTGT
M2-3-bottom	ATCTATCCACCTCCACCTCTACACCCATATCCATTCTATCATACCATCTTCAA
M probe-B_3 top	GGTATGATAGAATGGATATGGGTGT
M probe-B_4 bottom	ATCTATCCACCTCCACCTCTACACCCCTCATCTCATCATACACTTCCACAATC
M probe-B_4 top	AAGTGTATGATGAGATGAGGGGTGT
L_3	AAAGGGAGGT TGTACACATGGCTTCCA
C	TGTTGGATATTGGAAGCCATGTGTACATTTTTTTGATTGGAGTGGAGAGTTG
Input c	CGCGTGTAAACATCCCCGACTGGAAGCCATGTGTACAACCTCCCTTT) CTCGACCTCCTCTACCC
M probe-C bottom	TGTACACATGGCTTCCAATATCCAACACCTCTACCTCACTCACCTCATCACTA
M probe-C top	TAGAGGTGTTGGATATTGGAAGCCA
L_4	CGACGAGTGAGTGGAGAGAGATAGGTATGTCTACCCTGATCATACATCTACAT AGACTAGACGTGACCTACGCAGCACTGA
D_1	AGGGTAGACATACCTATCTCTCTCCACTTTTTTAGTAGTAAGTATGTTAGGT
D_2	ACGTCTAGTCTATGTAGATGTATGATCTTTTTAGATATGTATATTGAAAGTA
D_3	TAGATGATAGTCAGTGCTGCGTAGGTCTTTTTGATAGATAAGTAAGTGATGA
Input d	GGCCCTTTCAGTCGGATGTTTGCAGCTACCTATCTCTCTCCACTCACTCGTCGTC CTCTCTCTC
H_{d2}	TATGTAGATGTATGATCAGGGTAGACA
H_{d3}	TATGTAGATGTATGATCAGGGTAGACA
M probe-D_1 bottom	GTGGAGAGAGATAGGTATGTCTACCCTCCTCTACCTCACTCACCTCATCACTA
M probe-D_1 top	TAGAGGAGGGTAGACATACCTATCT
M probe-D_2 bottom	GATCATACATCTACATAGACTAGACGTCCTCTACCTCACTCACCTCATCACTA
M probe-D_2 top	TAGAGGACGTCTAGTCTATGTAGAT
M probe-D_3 bottom	GACCTACGCAGCACTGACTATCATCTACCTCTACCTCACTCACCTCATCACTA
M probe-D_3 top	TAGAGGTAGATGATAGTCAGTGCTG
S probe-E bottom (E probe)	ACAACCACTTACTTCTTCATCTATCCACCTCCACCTCT
S probe-E top	GGGTGTAGAGGTGGAGGTGGATAGAT
S probe-F bottom (F probe)	CCTCACTCACCT CATCACTACTATCATCACACATCTAT
S probe-F top	TAGTGATGAGGTGAGTGAGGTAGAGG
N probe bottom	GGATAGATGAAGAAGTAAGTGGTTGTTAGATGTGTGATGATAG TAGTGATG
N probe top	CTATCATCACACATCTATACAACCACTTACTTCTTC
RE bottom	ATGATGAGTGAGAGTGATGTTTTTTCATCCAATCACATACATCTCAACAACCAC TTACTTCTTC
RE top	ATAGATGAAGAAGTAAGTGGTTGTTGAGAT
Fuel E	TCTCAACAACCACTTACTTCTTC
RF bottom	CTATCATCACACATCTATTATGTACATAACACAATCACATTTTTGAGGATTGAG TGAGTGTAGA
RF top	TACATAATAGATGTGTGATGATAGTAGTGA
Fuel F	CTATCATCACACATCTATTATGT
FAM reporter bottom	TGAGATGTATGTGATTGGATG-FAM
FAM reporter top	BHQ1- CATCCAATCACATAC

ROX reporter bottom	ROX-TGTGATTGTGTTATGTACATA
ROX reporter top	BHQ2-CATAACACAATCACA
M reporter 1 bottom	GGGTGTAGAGGTGGAGGT-FAM
M reporter 1 top	BHQ1-ACCTCCACCTCT
M reporter 2 bottom	FAM-AGGTGAGTGAGGTAGAGG
M reporter 2 top	CCTCACTCACCTCA-BHQ1
S reporter 1 bottom	GGATAGATGAAGAAGTAAGTGGTTGT-FAM
S reporter 1 top	BHQ1-ACAACCACTTACTTCTTC
S reporter 2 bottom	ROX-ATAGATGTGTGATGATAGTAGTGATG
S reporter 2 top	CTATCATCACACATCTAT-BHQ2
miR-148a-3p	UCAGUGCACUACAGAACUUUGU
miR-182-5p	UUUGGCAAUGGUAGAACUCACACU
miR-30d-5p	UGUAAACAUCCCCGACUGGAAG
miR-30a-3p	CUUUCAGUCGGAUGUUUGCAGC

Supplementary Note 5

The rectangular origami was prepared according to the reported work[10]. The extended staples for anchoring computing probes and reporting probes are listed below:

Supplementary Table 5. Sequences of staples for the DNA origami framework.

Name	Sequence (5′ to 3′)
213 for A_1	CTCCCTACCCATATCTCATTTAAATATTTTTTGGAAGAAAAATCTACGACCAGTCA
196 for A_2	ACTAACTCCTACCCACACCTACTGGATAACGGAACAACATTATTACCTTATG
148 for A_1	CTCCCTACCCATATCTCATTCCAAAATATAATGCAGATACATAAACACCAGA
7 for A_2	ACTAACTCCTACCCACACCTCATAACCCGAGGCATAGTAAGAGCTTTTTAAG
13 for A_1	CTCCCTACCCATATCTCATTATCGGCTGCGAGCATGTAGAAACCTATCATAT
39 for A_2	ACTAACTCCTACCCACACCTGGTATTAAGAACAAGAAAAATAATTAAAGCCA
87 for A_1	CTCCCTACCCATATCTCATTTCATTACCCGACAATAAACAACATATTTAGGC
106 for A_2	ACTAACTCCTACCCACACCTTATAGAAGTTTTCGACAAAAGGTAAAGTAGAGAATA
204 for B_1	ATGGTAGCGTCCAGTAGCGCACGTTAGTAAATGAATTTTCTGTAAGCGGAGT
180 for B_2	GTTCCTCTAAACACGTTGGGCGTAACGATCTAAAGTTTTGTCGTGAATTGCG
156 for B_3	GATTGCTTGGGTCTCAGACATGTAGCATTCCACAGACAGCCCTCATCTCCAA
132 for B_4	GACGGGTAACGCGGACATTCTGAGTTTCGTCACCAGTACAAACTTAATTGTA
28 for B_1	ATGGTAGCGTCCAGTAGCGCCTCAGAGCCACCACCCTCATTTTCCTATTATT
52 for B_2	GTTCCTCTAAACACGTTGGGCCCTCAGAACCGCCACCCTCAGAACTGAGACT
76 for B_3	GATTGCTTGGGTCTCAGACATATCACCGTACTCAGGAGGTTTAGCGGGGTTT
100 for B_4	GACGGGTAACGCGGACATTCTTTTTATAAGTATAGCCCGGCCGTCGAG
10 for B_1	ATGGTAGCGTCCAGTAGCGCGCAATAGCGCAGATAGCCGAACAATTCAACCG
36 for B_2	GTTCCTCTAAACACGTTGGGGCCCAATACCGAGGAAACGCAATAGGTTTACC
60 for B_3	GATTGCTTGGGTCTCAGACAATCAGAGAAAGAACTGGCATGATTTTATTTTG
84 for B_4	GACGGGTAACGCGGACATTCTGAACAAACAGTATGTTAGCAAACTAAAAGAA
193 for B_1	ATGGTAGCGTCCAGTAGCGCTGCAACTAAGCAATAAAGCCTCAGTTATGACC
169 for B_2	GTTCCTCTAAACACGTTGGGTCCATATACATACAGGCAAGGCAACTTTATTT
145 for B_3	GATTGCTTGGGTCTCAGACACGAGTAGAACTAATAGTAGTAGCAAACCCTCA
122 for B_4	GACGGGTAACGCGGACATTCTCGCAAATGGGGCGCGAGCTGAAATAATGTGT
191 for C	CTGTAATATTGCCTGAGAGTCTGGAAAACTAGCAACTCTCCACTCCAATCAA
41 for C	ACGCTCAAAATAAGAATAAACACCGTGAATTTCAACTCTCCACTCCAATCAA
113 for C	CCAGCAGGGGCAAAATCCCTTATAAAGCCGGCCAACTCTCCACTCCAATCAA
25 for C	GAACGTGGCGAGAAAGGAAGGGAACAAACTATCAACTCTCCACTCCAATCAA
167 for D_1	CAACGCAATTTTTGAGAGATCTACTGATAATCACCTAACATACTTACTACTA

143 for D_2	TATATTTTAGCTGATAAATTAATGTTGTATAATACTTTCAATATACATATCT
120 for D_3	AGGTAAAGAAATCACCATCAATATAATATTTTTCATCACTTACTTATCTATC
65 for D_1	CATATTTAGAAATACCGACCGTGTTACCTTTTACCTAACATACTTACTACTA
89 for D_2	AGAGGCATAATTTCATCTTCTGACTATAACTATACTTTCAATATACATATCT
107 for D_3	TAAAGTACTTTTCGCGAGAAAACTTTTTATCGCAAGTCATCACTTACTTATCTATC
183 for D_1	TGGTTTTTAACGTCAAAGGGCGAAGAACCATCACCTAACATACTTACTACTA
159 for D_2	AGCTGATTACAAGAGTCCACTATTGAGGTGCCTACTTTCAATATACATATCT
135 for D_3	GAGTTGCACGAGATAGGGTTGAGTAAGGGAGCTCATCACTTACTTATCTATC
49 for D_1	AGGCGGTCATTAGTCTTTAATGCGCAATATTAACCTAACATACTTACTACTA
73 for D_2	GCCACGCTATACGTGGCACAGACAACGCTCATTACTTTCAATATACATATCT
97 for D_3	CTAAAGCAAGATAGAACCCTTCTGAATCGTCTTCATCACTTACTTATCTATC
201 for RE	ACATCACTCTCACTCATCATACGGCTACTTACTTAGCCGGAACGCTGACCAA
199 for RE	ACATCACTCTCACTCATCATCTTTGAAAAGAACTGGCTCATTATTTAATAAA
177 for RE	ACATCACTCTCACTCATCATTTTCATGAAAATTGTGTCGAAATCTGTACAGA
175 for RE	ACATCACTCTCACTCATCATCCAGGCGCTTAATCATTGTGAATTACAGGTAG
153 for RE	ACATCACTCTCACTCATCATATACGTAAAAGTACAACGGAGATTTCATCAAG
151 for RE	ACATCACTCTCACTCATCATAGTAATCTTAAATTGGGCTTGAGAGAATACCA
129 for RE	ACATCACTCTCACTCATCATAAACGAAATGACCCCCAGCGATTATTCATTAC
127 for RE	ACATCACTCTCACTCATCATCCAAATCACTTGCCCTGACGAGAACGCCAAAA
4 for RE	ACATCACTCTCACTCATCATGAGCCGCCCCACCACCGGAACCGCGACGGAAA
6 for RE	ACATCACTCTCACTCATCATTTATTCATAGGGAAGGTAAATATTCATTCAGT
31 for RE	ACATCACTCTCACTCATCATGCCACCACTCTTTTCATAATCAAACCGTCACC
33 for RE	ACATCACTCTCACTCATCATGACTTGAGAGACAAAAGGGCGACAAGTTACCA
55 for RE	ACATCACTCTCACTCATCATCACCAGAGTTCGGTCATAGCCCCCGCCAGCAA
57 for RE	ACATCACTCTCACTCATCATAATCACCAAATAGAAAATTCATATATAACGGA
79 for RE	ACATCACTCTCACTCATCATTGAGGCAGGCGTCAGACTGTAGCGTAGCAAGG
81 for RE	ACATCACTCTCACTCATCATCCGGAAACACACCACGGAATAAGTAAGACTCC
102 for RE	ACATCACTCTCACTCATCATACAAACAATTTTAATCAGTAGCGACAGATCGATAGC
103 for RE	ACATCACTCTCACTCATCATAGCACCGTTTTTTAAAGGTGGCAACATAGTAGAAAA
207 for RF	GGGAGAGGTTTTTGTAAAACGACGGCCATTCCCAGTTCTACACTCACTCAATCCTC
206 for RF	TATCAGGGTTTTCGGTTTGCGTATTGGGAACGCGCGTCTACACTCACTCAATCCTC
184 for RF	GCCAGCTGCCTGCAGGTCGACTCTGCAAGGCGTCTACACTCACTCAATCCTC
182 for RF	TGGACTCCCTTTTCACCAGTGAGACCTGTCGTTCTACACTCACTCAATCCTC
160 for RF	ACTGCCCGCCGAGCTCGAATTCGTTATTACGCTCTACACTCACTCAATCCTC
158 for RF	AGTTTGGAGCCCTTCACCGCCTGGTTGCGCTCTCTACACTCACTCAATCCTC

136 for RF	GTGAGCTAGTTTCCTGTGTGAAATTTGGGAAGTCTACACTCACTCAATCCTC
134 for RF	GAATAGCCGCAAGCGGTCCACGCTCCTAATGATCTACACTCACTCAATCCTC
114 for RF	GCATAAAGTTCCACACAACATACGAAGCGCCATCTACACTCACTCAATCCTC
112 for RF	CCGAAATCCGAAAATCCTGTTTGAAGCCGGAATCTACACTCACTCAATCCTC
23 for RF	GGATTTAGCGTATTAAATCCTTTGTTTTCAGGTCTACACTCACTCAATCCTC
26 for RF	TAGCCCTACCAGCAGAAGATAAAAACATTTGATCTACACTCACTCAATCCTC
48 for RF	AGATTAGATTTAAAAGTTTGAGTACACGTAAATCTACACTCACTCAATCCTC
50 for RF	GAATGGCTAGTATTAACACCGCCTCAACTAATTCTACACTCACTCAATCCTC
72 for RF	CTAAAATAGAACAAAGAAACCACCAGGGTTAGTCTACACTCACTCAATCCTC
74 for RF	GCGTAAGAGAGAGCCAGCAGCAAAAAGGTTATTCTACACTCACTCAATCCTC
96 for RF	ATCAACAGTCATCATATTCCTGATTGATTGTTTCTACACTCACTCAATCCTC
98 for RF	GCCAACAGTCACCTTGCTGAACCTGTTGGCAATCTACACTCACTCAATCCTC

S5 Reaction conditions.

Supplementary Table 6. Detailed reaction conditions used in this study.

**Probe preparation**		
Probe	Concentration	
L_n-N	L_1-A	$L_1/A_1/A_2$ = 10 µM/10 µM/10 µM
	L_2-B	$L_2/B_1/B_2/B_3/B_4$ = 10 µM/10 µM/10 µM/10 µM/10 µM
	L_3-C	L_3/C = 10 µM/10 µM
	L_4-D	$L_4/D_1/D_2/D_3$ = 10 µM/10 µM/10 µM/10 µM
DNA duplexes	M probe-A_1	top/bottom = 24 µM/20 µM
	M probe-A_2	top/bottom = 24 µM/20 µM
	M probe-B_1	top/bottom = 24 µM/20 µM
	M probe-B_2	top/bottom = 24 µM/20 µM
	M probe-B_3	top/bottom = 24 µM/20 µM
	M probe-B_4	top/bottom = 24 µM/20 µM
	M probe-C	top/bottom = 24 µM/20 µM
	M probe-D_1	top/bottom = 24 µM/20 µM
	M probe-D_2	top/bottom = 24 µM/20 µM
	M probe-D_3	top/bottom = 24 µM/20 µM
	S probe-E	top/bottom = 24 µM/20 µM
	S probe-F	top/bottom = 24 µM/20 µM
	N probe	top/bottom = 24 µM/20 µM
	RE probe	top/bottom = 24 µM/20 µM
	RF probe	top/bottom = 24 µM/20 µM
	Reporter	top/bottom = 24 µM/20 µM
**DNA origami preparation**		
DNA origami	M13 scaffold/staples = 10 nM/50 nM	
**DNA IC-CLA preparation**		
DNA IC-CLA	DNA origami/L_1-A/L_2-B/L_3-C/L_4-D/RE probe/RF probe = 1/2/2/2/2/2/2	
**Fluorescence kinetic experiment**		
Name	Concentration	Reaction condition
DNA IC-CLA	5 nM	Buffer: 1× TAE/Mg^{2+} buffer (1× Tris-acetate-EDTA, 10 mM $MgCl_2$)<br>Temperature: 37°C<br>Excitation/Emission wavelengths: 492/518 nm for FAM, 585/615 nm for ROX.<br>Multiplication: 5 nM of DNA IC-CLA, 25 nM of each H probe, 25 nM of each M probe, 200 nM of each M reporter, and 20 nM of each DNA input.<br>Summation: 5 nM of DNA IC-CLA or 20 nM of each Ln-N, 25 nM of each H probe, 25 nM of each M probe, 150 nM of each S probe, 200 nM of each S reporter, and 20 nM of each DNA input.<br>Subtraction: 5 nM of DNA IC-CLA, 200 nM of N probe, 200 nM of each Fuel probe (Fuel E and Fuel F), 200 nM
H_a	25 nM	
H_{b2}	25 nM	
H_{b3}	25 nM	
H_{b4}	25 nM	
H_{d2}	25 nM	
H_{d3}	25 nM	
M probe-A_1	25 nM	
M probe-A_2	25 nM	
M probe-B_1	25 nM	
M probe-B_2	25 nM	
M probe-B_3	25 nM	
M probe-B_4	25 nM	
M probe-C	25 nM	

M probe-D_1	25 nM	of each reporter, and different concentrations of E and F probes.<br>The entire computation: 5 nM of DNA IC-CLA, 25 nM of each H probe, 25 nM of each M probe, 150 nM of each S probe, 200 nM of N probe, 200 nM of each Fuel probe (Fuel E and Fuel F), 200 nM of each reporter, and varying combinations of DNA inputs or miRNA amplicon after LATE-PCR.
M probe-D_2	25 nM	
M probe-D_3	25 nM	
S probe-E	150 nM	
S probe-F	150 nM	
N probe	200 nM	
Fuel E	200 nM	
Fuel F	200 nM	
FAM reporter	200 nM	
ROX reporter	200 nM	

miRNA extraction

Total miRNA in serum samples (1 mL) was extracted using the Qiagen miRNeasy Serum/Plasma Kit according to the manufacturer's instructions. In brief, a lysis reagent was first added to lysis serum samples, then chloroform was added to separate the lysate. Subsequently, the samples underwent washing and elution process, and the eluted miRNA were stored in nuclease-free water at −80°C until needed.

Reverse transcription

Synthetic or extracted miRNAs were first reversely transcribed into cDNA by using a reverse transcription kit (Sangon) according to the manufacturer's instructions.

Components	Concentrations	Reaction condition
2×miRNA L-RT Solution mix	10 µL	16°C for 30 min → 37°C for 30 min → 85°C for 5 min → 4°C
miRNA L-RT Enzyme mix	1.5 µL	
miRNA	200 ng	
Stem-loop primer (10 µM)	1 µL	
RNase-free water	Up to 20 µL	

LATE-PCR

LATE-PCR was performed in PCR buffer containing template cDNA from reverse transcription, excess primer, limiting primer, TaqMan probe, 2× Hotstart PCR Master Mix (Sangon Biotech).

Components	Concentrations	Reaction condition
2×Hotstart PCR Master Mix	10 µL	95°C 5 min → 10 cycles of 95°C 10 sec, 55°C 10 sec and 72°C 20 sec → 40 cycles of 95°C 10 sec, 50°C 10 sec and 72°C 20 sec → 72°C 10 min → 4°C hold
Excess primer	1 µM	
Limiting primer	20 nM	
cDNA	As required	
PCR-grade water	Up to 20 µL	
TaqMan probes	As required	

Supplementary References

1. Genot, A. J., Zhang, D. Y., Bath, J. & Turberfield, A. J. Remote Toehold: A Mechanism for Flexible Control of DNA Hybridization Kinetics. *J. Am. Chem. Soc.* **133**, 2177-2182 (2011).
2. Zhang, D. Y. & Winfree, E. Control of DNA Strand Displacement Kinetics Using Toehold Exchange. *J. Am. Chem. Soc.* **131**, 17303-17314 (2009).
3. Tomczak, K., Czerwińska, P. & Wiznerowicz, M. ReviewThe Cancer Genome Atlas (TCGA): an immeasurable source of knowledge. *Contemp. Oncol. (Pozn).* **19**, A68-A77 (2015).
4. Cherry, K. M. & Qian, L. Scaling up molecular pattern recognition with DNA-based winner-take-all neural networks. *Nature* **559**, 370-376 (2018).
5. Qian, L., Winfree, E. & Bruck, J. Neural network computation with DNA strand displacement cascades. *Nature* **475**, 368-372 (2011).
6. Chatterjee, G., Dalchau, N., Muscat, R. A., Phillips, A. & Seelig, G. A spatially localized architecture for fast and modular DNA computing. *Nat. Nanotechnol.* **12**, 920-927 (2017).
7. Lopez, R., Wang, R. & Seelig, G. A molecular multi-gene classifier for disease diagnostics. *Nat. Chem.* **10**, 746-754 (2018).
8. Zhang, C. *et al.* Cancer diagnosis with DNA molecular computation. *Nat. Nanotechnol.* **15**, 709-715 (2020).
9. Okumura, S. *et al.* Nonlinear decision-making with enzymatic neural networks. *Nature* **610**, 496-501 (2022).
10. Chhabra, R. *et al.* Spatially addressable multiprotein nanoarrays templated by aptamer-tagged DNA nanoarchitectures. *J. Am. Chem. Soc.* **129**, 10304-10305 (2007).
