# Supplementary material for: A spatially localized DNA linear classifier for cancer diagnosis
Source: Nat Commun. 2024 May 29;15:4583. doi: 10.1038/s41467-024-48869-y (PMC11136972; doi:10.1038/s41467-024-48869-y)
Supplement: Supplementary file 3 — Reporting Summary [file 41467_2024_48869_MOESM3_ESM.pdf]

nature portfolio

Corresponding author(s): Da Han, Chao Zhang, Pei Guo

Last updated by author(s): May 4, 2024

Reporting Summary

Nature Portfolio wishes to improve the reproducibility of the work that we publish. This form provides structure for consistency and transparency in reporting. For further information on Nature Portfolio policies, see our Editorial Policies and the Editorial Policy Checklist.

Statistics

For all statistical analyses, confirm that the following items are present in the figure legend, table legend, main text, or Methods section.

n/a	Confirmed	
☐	☒	The exact sample size (n) for each experimental group/condition, given as a discrete number and unit of measurement
☐	☒	A statement on whether measurements were taken from distinct samples or whether the same sample was measured repeatedly
☐	☒	The statistical test(s) used AND whether they are one- or two-sided<br>*Only common tests should be described solely by name; describe more complex techniques in the Methods section.*
☒	☐	A description of all covariates tested
☒	☐	A description of any assumptions or corrections, such as tests of normality and adjustment for multiple comparisons
☐	☒	A full description of the statistical parameters including central tendency (e.g. means) or other basic estimates (e.g. regression coefficient) AND variation (e.g. standard deviation) or associated estimates of uncertainty (e.g. confidence intervals)
☐	☒	For null hypothesis testing, the test statistic (e.g. F, t, r) with confidence intervals, effect sizes, degrees of freedom and P value noted<br>*Give P values as exact values whenever suitable.*
☒	☐	For Bayesian analysis, information on the choice of priors and Markov chain Monte Carlo settings
☒	☐	For hierarchical and complex designs, identification of the appropriate level for tests and full reporting of outcomes
☒	☐	Estimates of effect sizes (e.g. Cohen's d, Pearson's r), indicating how they were calculated

Our web collection on statistics for biologists contains articles on many of the points above.

Software and code

Policy information about availability of computer code

Data collection	The Support Vector Machines (SVM) training and validation code used in this study is available on https://www.csie.ntu.edu.tw/~cjlin/libsvm/. The miRNA-seq data used in this study are available on TCGA database https://portal.gdc.cancer.gov.
Data analysis	The SVM training and validation was done using Matlab_R2018a. Data analysis was done using IBM SPSS Statistics V26 and Prism 8. Simulations were conducted with Python's SciPy package (version 1.2.1).

For manuscripts utilizing custom algorithms or software that are central to the research but not yet described in published literature, software must be made available to editors and reviewers. We strongly encourage code deposition in a community repository (e.g. GitHub). See the Nature Portfolio guidelines for submitting code & software for further information.

Data

Policy information about availability of data

All manuscripts must include a data availability statement. This statement should provide the following information, where applicable:

- Accession codes, unique identifiers, or web links for publicly available datasets
- A description of any restrictions on data availability
- For clinical datasets or third party data, please ensure that the statement adheres to our policy

All data supporting the results of this study are available within the paper and its Supplementary Information. The raw data are available from the corresponding authors upon request. Source data are provided with this paper. The miRNA-seq data used in this study are available on TCGA database https://

portal.gdc.cancer.gov.

Research involving human participants, their data, or biological material

Policy information about studies with human participants or human data. See also policy information about sex, gender (identity/presentation), and sexual orientation and race, ethnicity and racism.

Reporting on sex and gender	NA
Reporting on race, ethnicity, or other socially relevant groupings	NA
Population characteristics	NA
Recruitment	NA
Ethics oversight	KY2023-062-B by the Ethics Committee at Renji Hospital, School of Medicine, Shanghai Jiao Tong University

Note that full information on the approval of the study protocol must also be provided in the manuscript.

Field-specific reporting

Please select the one below that is the best fit for your research. If you are not sure, read the appropriate sections before making your selection.

[x] Life sciences [ ] Behavioural & social sciences [ ] Ecological, evolutionary & environmental sciences

For a reference copy of the document with all sections, see nature.com/documents/nr-reporting-summary-flat.pdf

Life sciences study design

All studies must disclose on these points even when the disclosure is negative.

Sample size	No statistical methods were used to predetermine sample size. Sample size was determined by the number of cases available in the TCGA database mined. For the in silica SVM training and validation, we obtained all the available miRNA-seq data from TCGA database with a total of 915 NSCLC and 105 healthy samples. Among them, 900 NSCLC and 90 healthy samples were randomly divided into a training set and a validation set with a 7:3 ratio for construction of in silica model, and the remaining 15 NSCLC and 15 healthy samples were saved for experimental testing with synthetic miRNA profiles replicated from those samples. For the evaluation of diagnostic accuracy of the DNA computation-based method, we used 50 clinical serum samples from 25 healthy and 25 NSCLC individuals. Herein, the sample size was largely determined by sample availability.
Data exclusions	No data was excluded from the analysis.
Replication	We replicated miRNA extraction, PCR, and fluorescent reporting at least 3 times across different conditions. We did not find any case where the data was not able to be replicated.
Randomization	NSCLC patients and healthy controls were enrolled in the study based on either confirmed diagnosis of non-small-cell lung cancer or health screening reports with no issue of the lungs. Therefore, randomization of individuals in different groups is not applicable.
Blinding	No blinding was performed. For training and validation of SVM models, we obtained all the available miRNA-seq data from TCGA database . For the clinical samples, we collected known types of samples from hospital according to the experimental needs to verify the accuracy of DNA molecular computation.

Reporting for specific materials, systems and methods

We require information from authors about some types of materials, experimental systems and methods used in many studies. Here, indicate whether each material, system or method listed is relevant to your study. If you are not sure if a list item applies to your research, read the appropriate section before selecting a response.

Materials & experimental systems

n/a	Involved in the study
☒	☐ Antibodies
☒	☐ Eukaryotic cell lines
☒	☐ Palaeontology and archaeology
☒	☐ Animals and other organisms
☐	☒ Clinical data
☒	☐ Dual use research of concern
☒	☐ Plants

Methods

n/a	Involved in the study
☒	☐ ChIP-seq
☒	☐ Flow cytometry
☒	☐ MRI-based neuroimaging

Clinical data

Policy information about clinical studies

All manuscripts should comply with the ICMJEguidelines for publication of clinical research and a completedCONSORT checklist must be included with all submissions.

Clinical trial registration	All serum samples were collected from Renji Hospital (Shanghai, China) with informed consent and approved with the registration ID of KY2023-062-B by the Ethics Committee at Renji Hospital, School of Medicine, Shanghai Jiao Tong University.
Study protocol	Study participants include non-small cell lung cancer (NSCLC) patients and healthy individuals. NSCLC samples were from the patients of the first diagnosis without any treatment. Healthy samples were from individuals with no issue of lungs reported in physical examination. The age, gender and cancer stage of all samples were not limited. Detailed clinical sample information is provided in Supplementary Table 2.
Data collection	Clinical information of patients and healthy individuals were collected by the hospital within last one year.
Outcomes	NA
